# Beauvericin suppresses the proliferation and pulmonary metastasis of osteosarcoma by selectively inhibiting TGFBR2 pathway

**DOI:** 10.7150/ijbs.86214

**Published:** 2023-08-21

**Authors:** Geni Ye, Yubo Jiao, Lijuan Deng, Minjing Cheng, Sheng Wang, Junqiu Zhang, Jie Ouyang, Yong Li, Yuxin He, Zhengchao Tu, Zhen Wang, Xiaojuan Song, Chenran Wang, Qi Qi, Dongmei Zhang, Lei Wang, Maohua Huang, Wencai Ye, Minfeng Chen

**Affiliations:** 1 State Key Laboratory of Bioactive Molecules and Druggability Assessment, Jinan University, Guangzhou, 510632, China.; 2 Guangdong Province Key Laboratory of Pharmacodynamic Constituents of Traditional Chinese Medicine and New Drugs Research, College of Pharmacy, Jinan University, Guangzhou, 510632, China.; 3 Guangzhou Key Laboratory of Formula-Pattern of Traditional Chinese Medicine, Jinan University, Guangzhou, 510632, China.; 4 MOE Key Laboratory of Tumor Molecular Biology, Clinical Translational Center for Targeted Drug, Department of Pharmacology, School of Medicine, Jinan University, Guangzhou, 510632, China.; 5 State Key Laboratory of Chemical Oncogenomics, Guangdong Provincial Key Laboratory of Chemical Biology, Tsinghua Shenzhen International Graduate School, Shenzhen, 518055, China.

**Keywords:** beauvericin, osteosarcoma, proliferation, pulmonary metastasis, TGF-β, TGFBR2

## Abstract

Osteosarcoma (OS) patients, particularly those with distant metastasis, experience rapid progression and derive poor survival benefits from traditional therapies. Currently, effective drugs for treating patients with metastatic OS remain scarce. Here, we found that the cyclic hexadepsipeptide beauvericin (BEA) functioned as a new selective TGFBR2 inhibitor with potent antiproliferative and antimetastatic activities against OS cells*.* Functionally, BEA inhibited TGF-β signaling-mediated proliferation, invasiveness, mesenchymal phenotype, and extracellular matrix remodeling of OS cells, and suppressed tumor growth and reduced pulmonary metastasis *in vivo*. Mechanistic investigation revealed that BEA selectively and directly bound to Asn 332 of TGFBR2 and inhibited its kinase activity, thereby suppressing the aggressive progression of OS cells. Together, our study identifies an innovative and natural selective TGFBR2 inhibitor with effective antineoplastic activity against metastatic OS and demonstrates that targeting TGFBR2 could be a potential therapeutic strategy for metastatic OS.

## Introduction

Osteosarcoma (OS), the most prevalent type of primary bone tumor, is often diagnosed in children and adolescents 1, 2. Due to high invasive and metastatic capacities, a large proportion of OS patients have clinically detectable metastases at initial diagnosis, and most patients with metastatic OS derive poor survival benefits from current therapeutic approaches, with a 5-year survival rate of about 20% 1, 3-5. Lung metastasis is the leading cause of cancer-related death in OS patients. The efficacy of oncogenic kinase-targeted therapies in OS patients is modest and limited due to the high levels of genomic instability in OS cells 1, 6, 7, and these drugs have not been approved for the treatment of OS. Currently, effective drugs for treating OS patients with distant metastasis remain lacking. Hence, there is an urgent need to develop innovative and more effective therapeutic agents for patients with metastatic OS.

The transforming growth factor-β (TGF-β) pathway is frequently activated in many types of cancer and significantly contributes to growth and metastasis 8, including OS 9, 10. Clinical investigation revealed that TGF-β levels were much higher in serum from OS patients with metastasis compared with those without metastasis 11; thus, the TGF-β signaling pathway may be considered as a therapeutic target in metastatic OS 10. Epithelial-mesenchymal transition (EMT) is characterized by elevated expression of mesenchymal markers, but loss of epithelial phenotype in cancer cells 12. Extracellular matrix (ECM) remodeling 13, 14, particularly alterations in collagen components, can be regulated by EMT-related transcription factors 15, 16. They are two biological processes closely associated with increased tumor invasiveness and metastasis, and are involved in the TGF-β-mediated invasiveness and distant metastasis of OS cells 17-19. Thus, targeting the TGF-β pathway may be a potential therapeutic option for metastatic OS.

Current inhibitors of TGF-β signaling are mainly selective TGFBR1 antagonists, but none of these inhibitors have been approved for treating OS, probably due to their poor efficacy. Most studies have investigated the role of TGF-β signaling in tumor progression and immunoregulation using mice with *Tgfbr2* depletion or expression of a dominant-negative form of *Tgfbr2*, which results in abrogation of any signal transduction induced by all three isoforms of TGF-β 20-23. Notably, a Cancer Cell Line Encyclopedia dataset revealed that the expression of TGFBR2 in OS cells was much higher than that of TGFBR1. Therefore, targeting TGFBR2 may be a more feasible strategy to inhibit the proliferation and metastatic spread of OS cells; however, effective and selective inhibitors of TGFBR2 are still scarce.

Beauvericin (BEA), a cyclic hexadepsipeptide, is one of the active ingredients of the traditional Chinese medicine *Bombyx batryticatus*. Accumulating studies have shown that BEA exerts a broad spectrum of biological effects, such as antimicrobial and anti-inflammatory effects 24, and has an immunostimulatory effect on dendritic cells 25. Studies have also revealed that BEA shows extensive anticancer effects and inhibits the progression of many types of cancer, such as colorectal cancer 26, pancreatic cancer 27, and non-small cell lung cancer 28. The antineoplastic mechanisms of BEA are probably associated with elevated intracellular Ca^2+^ levels and cell death 24, which still remain elusive. However, whether BEA exerts an antineoplastic effect on OS cells and the underlying mechanisms of BEA in inhibiting the malignant behavior and phenotype of OS are largely unknown.

Here, we found that BEA suppressed the proliferation and lung metastasis of OS cells by blocking the TGF-β/TGFBR2/Smad2/3 pathway, an effect possibly attributed to a direct interaction between BEA and Asn 332 of TGFBR2. Our study sheds new light on the molecular mechanisms of BEA in inhibiting malignant behavior and phenotype in OS cells and demonstrates that BEA has the potential to be developed as a promising agent for treating metastatic OS.

## Methods

### Materials and reagents

BEA with a purity of 98% was isolated from *Bombyx batryticatus* and dissolved in DMSO and stored at -20°C. The antibodies against Smad2/3 (8685), p-Smad2 (Ser465/467)/Smad3 (Ser423/425) (8828), N-Cadherin (13115), E-Cadherin (14472), Vimentin (5741), Slug (9585), ZEB1 (70512), FAK (71433), p-FAK (Tyr397) (8556), β-actin (#4970), Caspase-3 (14220), Cleaved Caspase-3 (9664), His-Tag (12698), and HA-Tag (5017) were obtained from Cell Singling Technology (Danvers, MA, USA). The antibodies against TGFRB2 (AF5449), p-TGFRB2 (Tyr284) (AF8191), TGFRB1 (AF5347), and p-TGFBR1 (Ser165) (AF8080) were purchased from Affinity Biosciences (Jiangsu, China). The antibodies against MMP2 (ab86607), COL1A1 (ab88147), COL3A1 (ab184993), and TGF-β1 (ab215715) were purchased from Abcam (Cambridge, MA, USA). Anti-mouse IgG, HRP-linked antibody (7076) and Anti-rabbit IgG, HRP-linked antibody (7074) were used as secondary antibodies and obtained from Cell Singling Technology (Danvers, MA, USA). SB431542 were obtained from TargetMol (T1726, Boston, MA, USA). MTT (ST316), DAPI (C1002), and 0.1% crystal violet solution (C0121) were purchased from Beyotime Biotechnology (Shanghai, China). 4% paraformaldehyde (G1101) was obtained from Servicebio (Wuhan, China).

### Cells and cell culture

Human OS 143B and SJSA-1 cells were purchased from the American Type Culture Collection (ATCC; Rockville, MD, USA), and U2OS cells were obtained from the Cell Bank of Chinese Academy of Sciences (Shanghai, China). They were cultured in DMEM (11965092, Gibco, Grand Island, NY, USA) containing 10% fetal bovine serum (FBS; FSP500, ExCell Bio, Shanghai, China) and 1% penicillin-streptomycin (15140122, Gibco) and maintained in a humidified environment supplemented with 5% CO_2_ at 37°C. Human immortalized hFOB 1.19 osteoblasts (Procell Life Science & Technology Co., Ltd., Wuhan, China) were cultured in osteoblast medium (CM-0533, Procell) containing 0.3 mg/mL G-418 disulfate (T6512, TargetMol) at 34°C. All cells were confirmed negative for mycoplasma.

### Cell viability and colony formation assays

An MTT assay was performed to evaluate the effect of BEA on the viability of OS cells and hFOB 1.19 osteoblasts 29, and the effect of BEA on the clonogenicity of OS cells was determined by a cell colony formation assay as previously described 30.

### Wound healing, cell migration, and invasion assays

The effects of BEA on the migration and invasion capacities of OS cells were evaluated by wound healing 29 and Transwell migration and invasion assays 31 as described in our prior studies.

### Quantitative PCR (qPCR) analysis and Western blotting

The effects of BEA on the expression of EMT- and collagen deposition-related genes and Smad2/3 target genes in OS cells were measured by qPCR, and the effects of BEA on the expression of EMT- and collagen deposition-associated proteins and the target effector proteins of the TGF-β/Smad2/3 pathway were evaluated by Western blotting, which were performed as previously described 32, 33. The primer sequences are listed in **Table S1**.

### Phosphoproteomic analysis

143B cells treated with or without 3 μM BEA for 24 h, and phosphoproteomic analysis of OS cells was then performed by Shanghai Applied Protein Technology Co., Ltd. as described previously 34. Briefly, 143B cells (5 × 10^7^) after treatment with vehicle or BEA were washed twice with precooled PBS. SDT buffer (100 mM Tris-HCl, pH 7.6, 4% SDS, and 1 mM DTT) was then added to the dishes placed on ice, and the cells were quickly harvest by scraping, flash-frozen in liquid nitrogen, followed by storage at -80°C. The protein concentration was quantified using a Pierce™ BCA protein assay kit (23227, Thermo Scientific, Waltham, MA, USA). Protein was digested by trypsin, which was performed according to the Filter-Aided Sample Preparation (FASP) procedure that was previously described by Matthias Mann. Next, the digested peptides in each sample were desalted on C18 cartridges (Empore™ SPE Cartridges C18, standard density, bed I.D. 7 mm, Volume 3 mL, Sigma), concentrated by vacuum centrifugation, and reconstituted in 40 µL of 0.1% (*v*/*v*) formic acid. Enrichment of phosphopeptides was carried out using a High-Select^TM^ Fe-NTA Phosphopeptide Enrichment Kit (A32992, Thermo Scientific) according to the manufacturer's instructions. After lyophilization, the phosphopeptides were resuspended in 20 µL of loading buffer (0.1% formic acid). Finally, the samples were analyzed by LC/MS. The raw MS data of each sample were combined, followed by identification and quantitative analysis using MaxQuant software.

### Cell transfection

TGFBR2-HA (HG10358-CY) was purchased from Sino Biological (Sino Biological, Beijing, China). pCMV5B-TGFbeta receptor II K277R, kinase dead (TGFBR2-KD; #11762, https://www.addgene.org/11762/) was obtained from Addgene. TGFBR1-EGFP and TGFBR2 mutants (TGFBR2^N332D^, TGFBR2^N332T^, and TGFBR2^K252R^) were purchased from Qinda Biotech (Wuhan, China). OS cells were transfected with indicated plasmids or small interfering RNA (siRNA) duplexes targeting TGFRB2 using Lipofectamine™ 3000 (Invitrogen). The corresponding empty vectors and scrambled siRNAs served as negative controls. The sequences of TGFBR2 siRNA are listed in **Table S2**. After transfection for 6-8 h, the medium was changed, and the cells were continued to culture for 24 h. Next, transfected cells were further treated with TGF-β1 in the absence or presence of BEA, and *in vitro* experiments were performed.

#### Smad luciferase activity assay

Briefly, 2 μg of Smad luciferase reporter plasmid (11543ES03, Yeasen Biotechnology, Shanghai, China) was transfected into OS cells using Lipofectamine™ 3000 (Invitrogen). An equal amount of* Renilla* luciferase plasmid was transfected into 143B and U2OS cells and served as an internal control. After 24 h of transfection, TGF-β1 in the absence or presence of BEA was used to treat OS cells for another 24 h. The SMAD luciferase and *Renilla* luciferase signals of OS cells were evaluated using a Dual Luciferase Reporter Gene Assay Kit (RG027, Beyotime Biotechnology) according to the manufacturer's instructions.

### Cellular immunofluorescence assay

The effects of BEA on TGF‐β1-induced EMT and nuclear translocation of p-Smad2/3 in OS cells were assessed by an immunofluorescence assay. Briefly, OS cells were seeded in confocal culture dishes and cultured overnight, followed by treatment with BEA and TGF‐β1 for 24 h. Next, the cells were fixed with 4% paraformaldehyde, blocked, and permeabilized prior to incubation with antibodies against p-Smad2/3, E-cadherin, ZO-1, ZEB1, and Vimentin at 4°C overnight. The cells were then incubated with the corresponding secondary antibodies at room temperature in the dark for 1 h, and nuclei were stained with DAPI. The cells were observed and images were acquired with a confocal laser scanning microscope (Zeiss, LSM 800).

### *In vitro* kinase activity assay

The effect of BEA on the kinase activity of TGFBR2 and TGFBR1 was evaluated by time-resolved fluorescence resonance energy transfer (TR-FRET) with a LanthaScreen Eu Kinase Binding Assay. TGFBR2 and TGFBR1, Eu-anti-GST Antibody, and Kinase Tracer 178 were purchased from Life Technologies (Carlsbad, USA). All reactions were carried out in 384-well plates, with a reaction volume of 10 μL. The 5× kinase buffer was diluted 1× with deionized water, and TGFBR2, TGFBR1, and the EU-anti-GST antibody were diluted with 1× kinase buffer to the required concentrations. Five microliters of the 2× TGFBR2/EU-anti-GST antibody or TGFBR1/EU-anti-GST antibody mixture (HEPES, pH 7.5, 1 mM EGTA, 0.01% BRIJ-35, 10 mM MgCl_2_, 12 nM TGFBR2, 12 nM TGFBR1, and 4 nM antibody) was added to the 384-well plates. The Echo520 ultra-micro liquid pipetting system was used to transfer a series of 10 nL volumes of the mixtures (3-fold concentration gradient). The mixtures were incubated with shaking at room temperature for 10-20 min, and 5 μL of 2× Kinase Tracer 178 (120 nM) was added. Next, the mixtures were further incubated with shaking and centrifuged, and the reactions were allowed to proceed at room temperature in the dark for 1 h. An EnVision multilabel plate reader (PE Company) was used for detection, with an excitation wavelength of 340 nm and emission wavelengths of 665 nm and 615 nm. Based on the fluorescence ratio, the inhibition rate of the enzymatic reaction by BEA and the IC_50_ values of BEA for TRGBR2 and TGFBR1 were calculated.

### Molecular docking study

The X-ray crystal structure of transforming growth factor β receptor II (TGFBR2) with a co-crystallized ligand (PDB ID: 5E8Y) is available in the Protein Data Bank, and the docking of the co-crystallized ligand to the binding region of 5E8Y was performed using AutoDock Vina software. The value of root-mean-square deviation (RMSD) = 0.206 Å and that of binding energy score = -8.36 kcal mol^-1^ were considered a good cutoff for confirmation of computed ligand-protein docking. The target compound BEA was docked within the 5E8Y structure, and the outcome showed that BEA formed four H-bond interactions and H-bond lengths were recorded. The binding energy scores and 3D pose views were generated for further analysis of the interaction of BEA with TGFBR2 and the associated binding affinities.

### Drug affinity responsive target stability (DARTS) approach

The binding affinity of BEA for TGFBR2 and TGFBR1 was evaluated by DARTS approach. Briefly, 143B cells (2 × 10^6^) were lysed with immunoprecipitation lysis buffer (20 mM Tris-HCl pH 7.5, 2 mM EDTA, 150 mM NaCl, 1.5 mM MgCl_2_, and 0.5% NP-40) containing protease inhibitor cocktail (TargetMol). Following the determination of protein concentration, OS cell lysates were added to TNC buffer (50 mM Tris-HCl pH 8.0, 50 mM NaCl, and 10 mM CaCl_2_), incubated with various concentrations of BEA, SB431542, or DMSO (vehicle) at room temperature for 2 h, and digested with pronase (CP9141, Coolaber, Beijing, China) at room temperature for 30 min. Next, protein digestion was terminated with SDS-PAGE loading buffer, and proteins in the samples were denatured at 100°C for 10 min. Finally, the expression of target proteins was determined by Western blotting.

### Cellular thermal shift assay (CETSA)

OS cells were treated with BEA for 4 h, collected, and evenly divided into 8 fractions. After heating along a specific temperature gradient for 10 min, the fractions were subjected to 3 freeze-thaw cycles in liquid nitrogen, and the cell lysates were centrifuged at 12,000 rpm and 4°C for 12 min to obtain total protein extracts. Then, the total protein extracts were mixed with SDS-PAGE loading buffer and subjected to heat denaturation for Western blotting.

### Microscale thermophoresis (MST) assay

The binding of BEA to TGFBR2 was evaluated by an MST assay. Recombinant human TGFBR2 ECD (Sino Biological) labeled with a Monolith NT Protein Labeling Kit RED-NHS (Nanotemper, Munich, Germany) was diluted to 140 nM with PBS, and label-free BEA was diluted at half concentration (500,000 - 244 nM) with PBS containing 10% DMSO and 0.05% Tween-20. Next, the protein samples were mixed with BEA and incubated at room temperature for 5 min. The mixtures were loaded into capillary tubes, and the MST assay was performed using a Monolith NT.115 instrument (Nanotemper, Germany).

### Animal study

All animal studies were conducted with the approval of the Laboratory Animal Ethics Committee of Jinan University (approval number: 12327) and adhered to the NIH Guide for the Care and Use of Laboratory Animals. The antiproliferative and antimetastatic effects of BEA on OS cells *in vivo* were determined with an OS orthotopic xenograft mouse model as previously described 29. Male BALB/c nude mice (4 to 6 weeks old) were obtained from GemPharmatech Co., Ltd. (Nanjing, China), and housed in a specific pathogen free room at 22°C ± 2°C and 50% - 60% humidity, with a 12-h light/dark cycle and a standard rodent diet and water. The mice were anesthetized with isoflurane, and 143B cells (4 × 10^7^ cells/mL) suspended in serum-free DMEM (50 μL per mouse) were orthotopically injected with an insulin syringe into the proximal end of the tibia of each mouse. When tumors grew to about 75 mm^3^, the mice were grouped randomly and treated with vehicle or BEA (2.5 and 5 mg/kg, intravenous injection, once a day). The tumor volumes (V) were calculated with the following formula: V = 4/3 × π × [1/4 × (a + b)]^2^, and “a” indicates the longer diameter and “b” refers to the shorter diameter that is perpendicular to a. After treatment for 14 days, the mice were euthanized, and tumors were resected, weighed, and images of tumors were acquired. The xenograft tumors (containing tibias) were harvested and subjected to histological and immunohistochemical analyses. For the toxicity of BEA in tumor-bearing mice, the organs (heart, liver, spleen, lung, and kidney) of mice in each group were harvested and subjected to histological analysis. Serum samples of each mouse were harvested for the measurement of creatinine (CRE) and blood urea nitrogen (BUN) by ELISA.

### Histological and immunohistochemical analyses

Histological and immunohistochemical analyses of the OS xenograft tumors and organs from tumor-bearing mice were performed according to our prior studies 35, 36. For hematoxylin-eosin (H&E) staining, OS xenograft tumors and organs from each mouse were fixed, embedded in paraffin, sliced into 5-μm sections, and stained with H&E following standard procedures. The area and number of lung metastatic foci were analyzed and quantified in accordance with our prior study 29. For immunohistochemical (IHC) staining, the slides were subjected to deparaffinization, dehydration, and antigen retrieval, and were then incubated with the indicated primary antibodies at 4°C overnight. Next, the slides were incubated with the corresponding HRP-conjugated secondary antibodies, followed by staining with a DAB kit (G1212, Servicebio). The slides were observed, and images were acquired using an inverted microscope (IX70, Olympus). IHC staining of each protein in OS xenograft tumors was quantified with ImageJ software.

### Statistical analysis

Data are presented as mean ± standard error of mean (SEM). All *in vitro* experiments were performed at least three independent times and conducted in a blinded and randomized manner. Statistical analyses were performed using GraphPad Prism software (version 8.4.3). The significance of differences between two groups was analyzed using unpaired two-tailed *t*-test, and the significance of differences among more than two groups was determined by one-way ANOVA with Tukey's multiple comparison test. *P* < 0.05 indicated a statistically significant difference.

## Results

### BEA suppresses the viability, colony formation, and motility of OS cells *in vitro*

**Figure 1:**
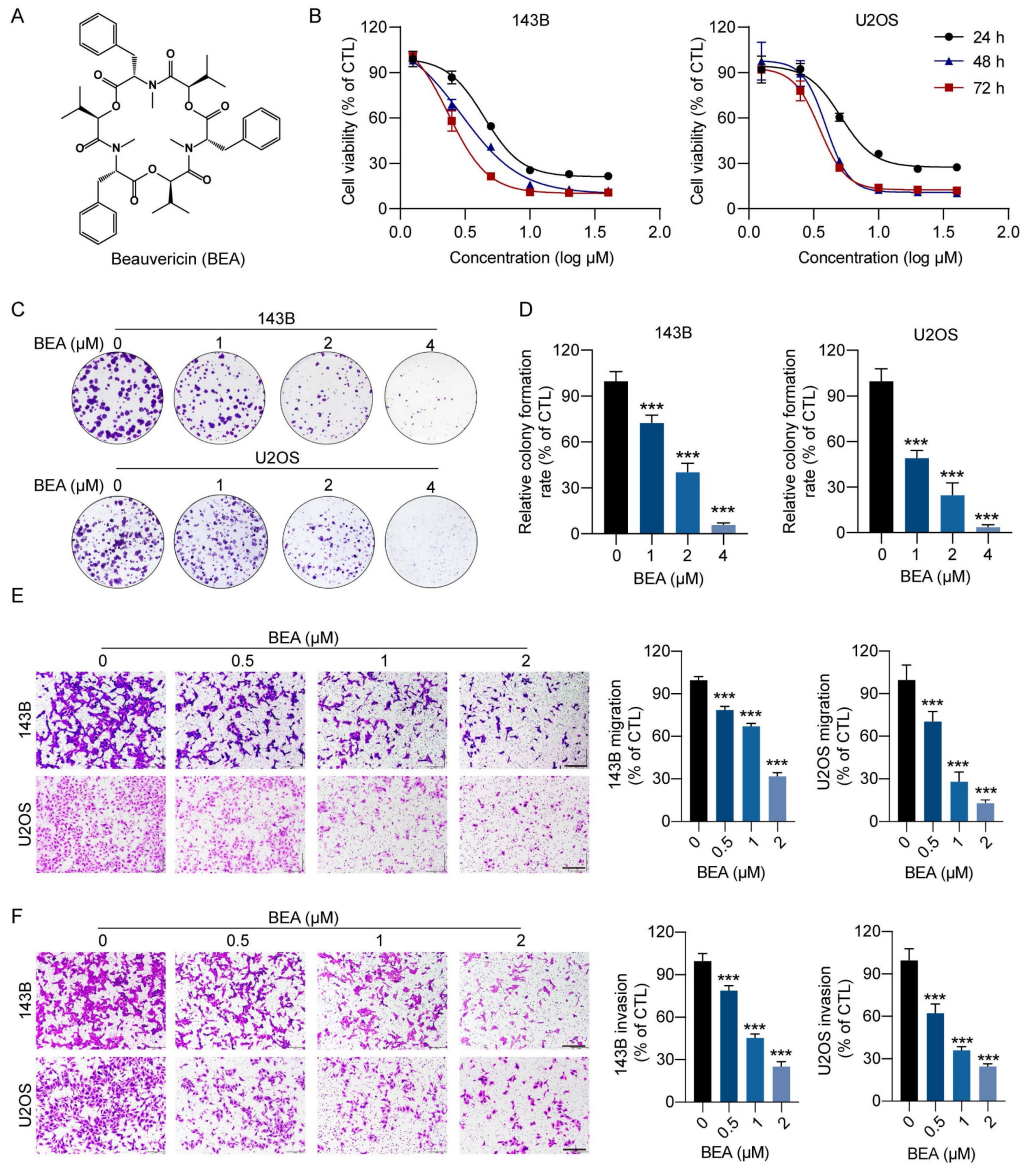
** BEA suppresses the proliferation and motility of OS cells *in vitro*.** (A) The chemical structure of BEA. (B) The effect of BEA on the viability of OS cells (143B and U2OS) was assessed by an MTT assay. (C and D) Colony formation assay of 143B and U2OS cells that were treated with the indicated concentrations of BEA. Representative images and quantification of cell colonies are shown. (E and F) The effect of BEA on the migration and invasion of OS cells was evaluated by Transwell (E) migration and (F) invasion assays. Representative images and quantification of the numbers of (E) migrated and (F) invaded OS cells are shown. Scale bar: 200 μm. Data are presented as mean ± SEM. *n* = 3.^ ***^ *p* < 0.001 *vs.* the CTL (control) group.

To search for innovative and effective agents for treating OS, more than 200 natural compounds were tested, and 143B cell viability was evaluated. We found that beauvericin (BEA), a cyclic hexadepsipeptide (**Figure 1A**), potently reduced the viability of 143B cells and was selected for the following studies. The effect of BEA on the viability and clonogenicity of OS cells were further assessed. Our results revealed that BEA dramatically decreased the viability of OS cells (143B, U2OS, and SJSA-1 cells) in a concentration- and time-dependent manner, with IC_50_ values (48 h) of 3.41 ± 0.08 μM in 143B cells, 3.88 ± 0.01 μM in U2OS cells, and 2.94 ± 0.07 μM in SJSA-1 cells (**Figure 1B and S1A-B**). However, BEA showed a lower ability to decrease the viability of human hFOB 1.19 osteoblasts than the viability of human OS cells (**Fig S1C**). Moreover, BEA treatment significantly decreased the clonogenicity of OS cells, as evaluated by a colony formation assay (**Figure 1C-D**). BEA also induced apoptosis in 143B cells (**Figure S1D**). High invasiveness and metastasis are aggressive behaviors of OS cells, and the effect of BEA on the migration and invasion of OS cells was investigated. We found that BEA inhibited the closure of wound in OS cell monolayers (**Figure S1E-F**) and dramatically decreased the migration and invasion capacities of 143B and U2OS cells in a concentration-dependent manner (**Figure 1E-F**). Together, these results reveal that BEA shows antiproliferative and antimetastatic effects on OS cells *in vitro*.

### BEA inhibits the TGF-β/Smad2/3 signaling pathway in OS cells

**Figure 2:**
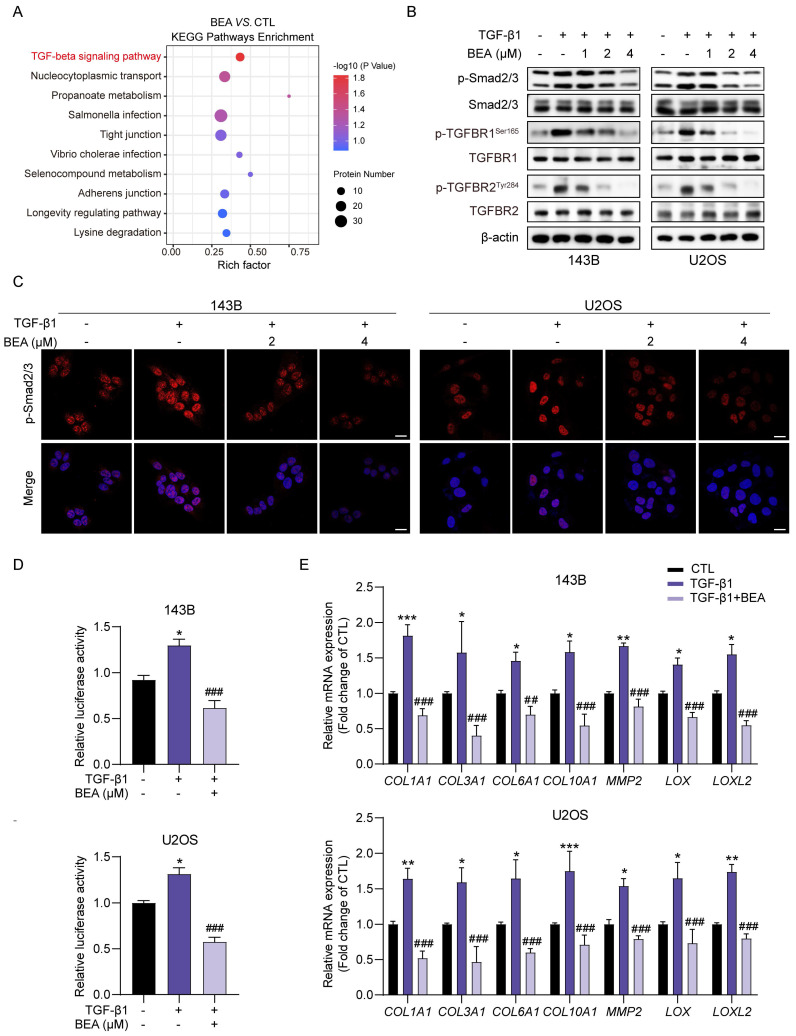
** BEA inhibits the TGF-β/Smad2/3 signaling pathway in OS cells.** (A) KEGG pathway enrichment analysis in BEA-treated and untreated 143B cells. (B) The effect of BEA on the TGF-β1-induced phosphorylation of Smad2/3, TGFBR1, and TGFBR2 in OS cells was evaluated by Western blotting. (C) Cellular immunofluorescence assay was performed to assess the nuclear translocation of p-Smad2/3 in OS cells treated with BEA. Scale bar: 20 μm. (D) The transcriptional activity of Smad2/3 in OS cells treated as indicated was determined by a Smad2/3 luciferase reporter assay. (E) 143B and U2OS cells were treated with BEA (2 μM) for 24 h and total RNA was harvested and analyzed by qPCR. Quantification of the mRNA levels of *COL1A1*, *COL3A1*, *COL6A1*, *COL10A1*, *MMP2*, *LOX*, *LOXL2* in OS cells. Data are presented as mean ± SEM. *n* = 3. ^*^ *p* < 0.05, ^**^ p < 0.01, and ^***^ *p* < 0.001 *vs.* the CTL (control) group, and ^##^* p* < 0.01 and ^###^ *p* < 0.001 *vs.* the TGF-β1-treated group.

To further investigate the mechanisms underlying the BEA-mediated inhibition of malignant behavior in OS cells, phosphoproteomic analysis of 143B cells treated with either vehicle or BEA was conducted. KEGG enrichment analysis indicated that TGF-beta signaling pathway may be associated with the antineoplastic effect of BEA on OS cells (**Figure 2A**). Due to the involvement of TGF-β pathway in the aggressive progression of OS cells, the effect of BEA on this axis was evaluated. Indeed, TGF-β1 stimulation promoted the phosphorylation of Smad2/3, TGFBR1**,** and TGFBR2 in 143B and U2OS cells, whereas these increases were dramatically suppressed in OS cells after treatment with BEA (**Figure 2B and S2**). Immunofluorescence staining revealed that BEA treatment markedly reduced the TGF-β1-induced nuclear translocation of p-Smad2/3 in OS cells (**Figure 2C**). Consistent with this finding, BEA treatment decreased the luciferase activity of Smad2/3 in 143B and U2OS cells treated with TGF-β1 (**Figure 2D**). Moreover, the TGF-β signaling pathway regulates extracellular matrix remodeling, characterized by elevated expression of collagens, matrix metalloproteinases, and proteins in lysyl oxidase family 14, 16, 19, in the tumor microenvironment. Our results showed that TGF-β incubation promoted the expression of various genes associated with ECM remodeling, including *COL1A1*, *COL3A1*, *COL6A1*, *COL10A1*, *MMP2*, *LOX*, and* LOXL2*, in OS cells, whereas these effects were almost completely abrogated by BEA treatment (**Figure 2E**). Collectively, these data demonstrate that BEA inhibits the activation of TGF-β/Smad2/3 signaling pathway in OS cells.

### BEA abrogates TGF-β1-mediated malignant behavior and aggressive phenotype in OS cells

**Figure 3:**
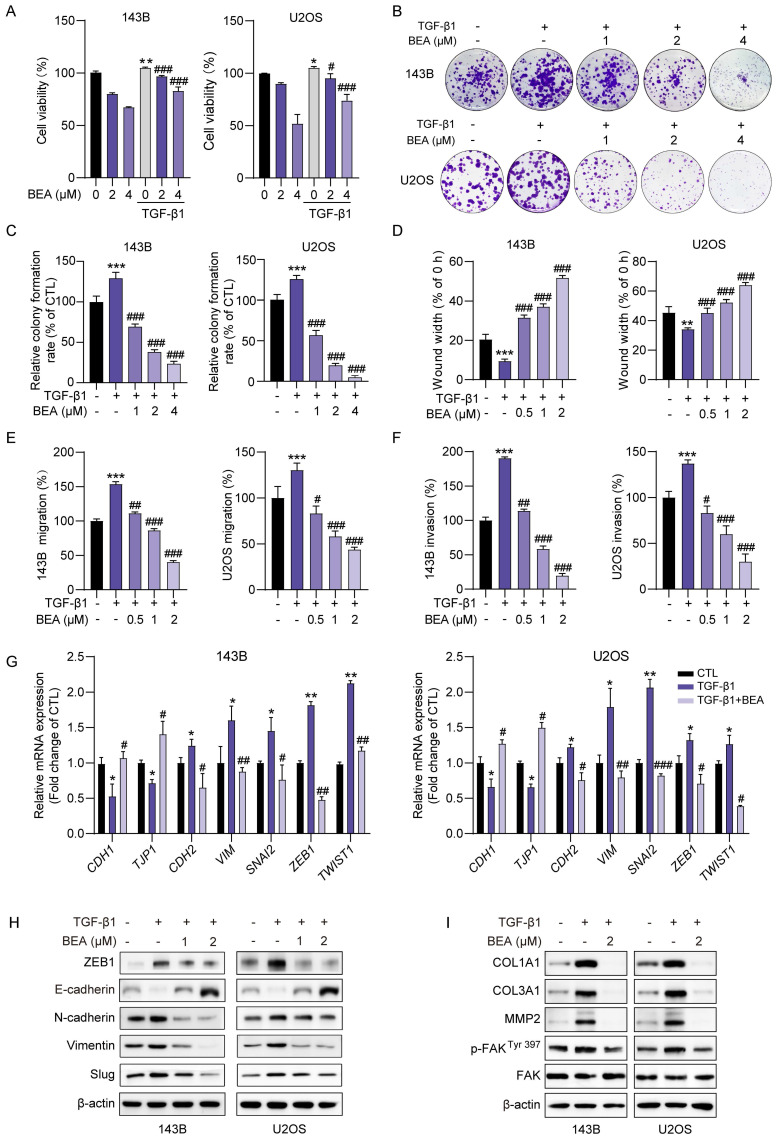
** BEA abrogates TGF-β1-mediated malignant behaviors and aggressive phenotypes in OS cells.** (A) The effect of BEA on the TGF-β1-induced viability of OS cells was evaluated by an MTT assay. (B and C) The effect of BEA on the TGF-β1-induced colony formation of OS cells was determined by a cell colony formation assay. Representative images and quantification of cell colonies are shown. (D) The effect of BEA on TGF-β1-induced migration in OS cells at 24 h was evaluated by a wound healing assay. (E and F) Transwell migration and invasion assays were performed to determine the effect of BEA on TGF-β1-induced migration and invasion in 143B and U2OS cells. (G) The mRNA expression of epithelial and mesenchymal markers in OS cells was measured by qPCR. (H) Western blot analysis of the effect of BEA on TGF-β1-induced an increase in mesenchymal phenotype in OS cells. (I) Western blotting for the effect of BEA on the TGF-β1-induced ECM remodeling. Data are presented as mean ± SEM. *n* = 3.^ *^ p < 0.05, ^**^ p < 0.01, ^***^ p < 0.001 *vs.* the CTL (control) group, and ^#^* p* < 0.05, ^##^* p* < 0.01, ^###^* p* < 0.001 *vs.* the TGF-β1-treated group.

Since BEA inhibited the TGF-β1-induced phosphorylation and Smad2/3 transcriptional activity of OS cells, the malignant behavior and aggressive phenotype of OS cells treated with TGF-β1 and BEA were further investigated. We found that TGF-β1-mediated increase in viability in OS cells was suppressed by BEA treatment (**Figure 3A**). BEA treatment also dramatically decreased the TGF-β1-induced colony formation of 143B and U2OS cells (**Figure 3B-C**). Next, the effect of BEA on TGF-β1-induced OS cell migration and invasion was investigated. Our results revealed that incubation with TGF-β1 facilitated the closure of wound in OS cell monolayers, and that would closure was significantly inhibited after BEA treatment (**Figure 3D and S3A-B**). BEA treatment also dramatically suppressed the TGF-β1-induced migration of OS cells, and similar results were obtained in the Transwell invasion assay (**Figure 3E-F and S3C**). Additionally, TGF-β1 incubation increased mesenchymal phenotype in 143B and U2OS cells, whereas BEA treatment potently decreased the expression of mesenchymal markers in OS cells, as assessed by qPCR (**Figure 3G**), Western blotting (**Figure 3H and S4A**), and immunofluorescence staining (**Figure S4B**). For ECM remodeling, BEA treatment decreased the expression of COL1A1, COL3A1, MMP2, and phosphorylated FAK in TGF-β1-treated OS cells (**Figure 3I and S4C**). Taken together, these findings indicate that BEA suppress the TGF-β1-mediated proliferation, invasiveness, increased mesenchymal phenotype, and ECM remodeling in OS cells.

### BEA interacts with TGFBR2 and inhibits the TGFBR2-mediated aggressive progression of OS cells *in vitro*

**Figure 4:**
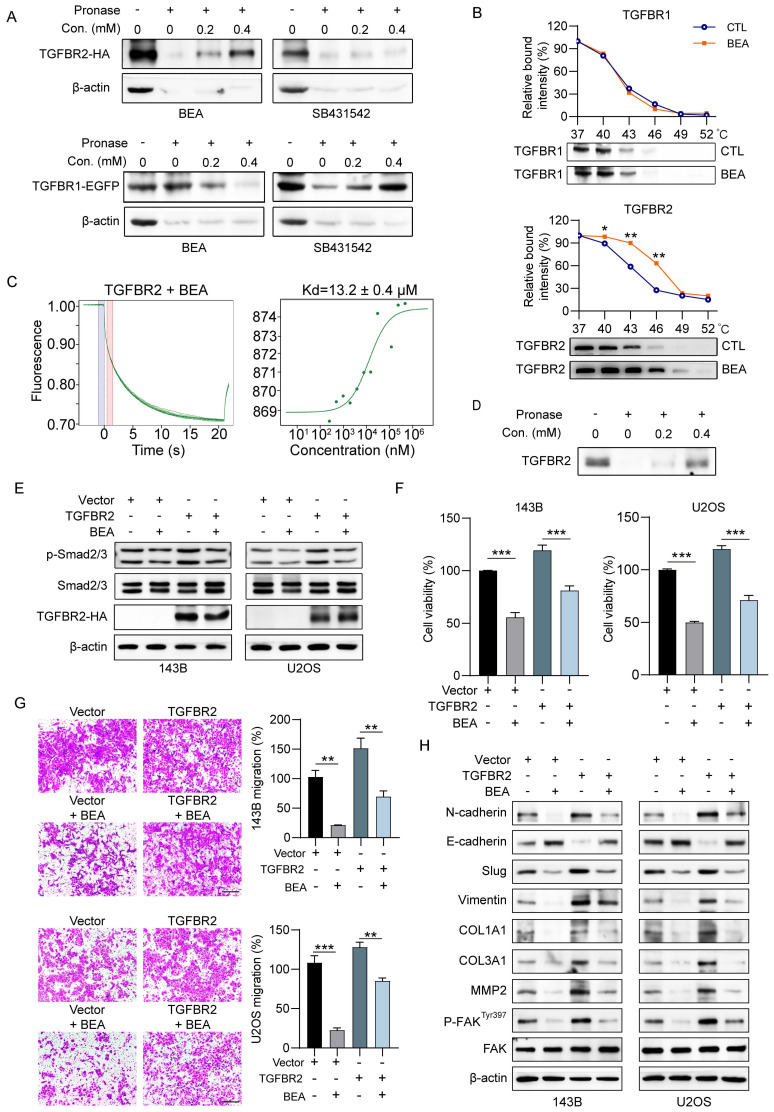
** BEA interacts with TGFBR2 and inhibits the TGFBR2-mediated aggressive progression of OS cells *in vitro*.** (A) The binding of BEA to TGFBR2 and TFGBR1 was determined by DARTS approach. SB431542 was used as the negative control for TGFBR2 and the positive control for TGFBR1. (B) CETSA was used to evaluate the binding of BEA to TGFBR2 and TGFBR1 in OS cells. (C) The binding between BEA and TGFBR2 was determined by an MST assay. (D) DARTS approach was used to evaluate the effect of BEA on the stability of recombinant human TGFBR2 protein. (E-H) After transfection with an TGFBR2 overexpression plasmid or vector, OS cells were treated with or without BEA. (E) Western blotting was conducted to evaluate the expression levels of p-Smad2/3, Smad2/3, and TGFBR2 in OS cells. (F) An MTT assay was performed to evaluate the effect of BEA on the viability of OS cells. (G) A Transwell migration assay was performed to evaluate the effect of BEA on the migration of OS cells. Scale bar: 200 μm. (H) Western blot analysis of the expression of epithelial and mesenchymal markers and ECM remodeling-associated proteins in OS cells after treatment with BEA. Data are presented as mean ± SEM. *n* = 3. ^*^ p < 0.05, ^**^ *p* < 0.01, and ^***^ *p* < 0.001*.*

Next, we investigated the underlying molecular mechanisms by which BEA suppresses the aggressive progression of OS cells and inhibits the interactions of BEA with TGFBR2 and TGFBR1. We found that BEA can protect TGFBR2 from pronase-mediated degradation in a concentration-dependent manner, whereas BEA failed to protect TGFBR1 from pronase-mediated degradation (**Figure 4A**). The melting curve of TGFBR2 was significant shifted in the presence of BEA compared with that of TGFBR1 (**Figure 4B**). These data indicate that BEA greatly stabilizes TGFBR2, but not TGFBR1, in OS cells. The MST assay results further confirmed a direct binding between BEA and TGFBR2 (**Figure 4C**). Additionally, since the direct binding of TGFBR2 to TGFBR1 is required for TGF-β pathway activation 37, DARTS approach was further performed with purified recombinant human TGFBR2. As expected, BEA incubation protected recombinant human TGFBR2 protein from pronase-mediated degradation (**Figure 4D**), suggesting that BEA can directly bind to and stabilize TGFBR2.

To further evaluate whether the antineoplastic effect of OS cells is regulated by TGFBR2, OS cells (143B and U2OS) were transfected with a TGFBR2 overexpression construct (**Figure S5A**). Similar to the effects induced by TGF-β1, TGFBR2 overexpression increased the phosphorylated levels of Smad2/3 in OS cells (**Figure 4E and S5B**), indicating that TGFBR2 overexpression activates the TGF-β pathway in OS cells. BEA treatment dramatically reduced the phosphorylation of Smad2/3 (**Figure 4E and S5B**) and decreased cell viability in TGFBR2-overexpressing OS cells (**Figure 4F**). BEA treatment also decreased the migration and invasion capacities of TGFBR2-overexpressing 143B and U2OS cells (**Figure 4G**). Additionally, TGFBR2 overexpression increased the expression of mesenchymal markers and collagen components in OS cells, whereas these enhancements were suppressed by BEA treatment (**Fig 4H and S5C**). Taken together, our results indicate that BEA selectively interacts with TGFBR2 and abrogates TGFBR2-induced malignant behavior and phenotype in OS cells.

### Modes and sites of binding between BEA and TGFBR2

**Figure 5:**
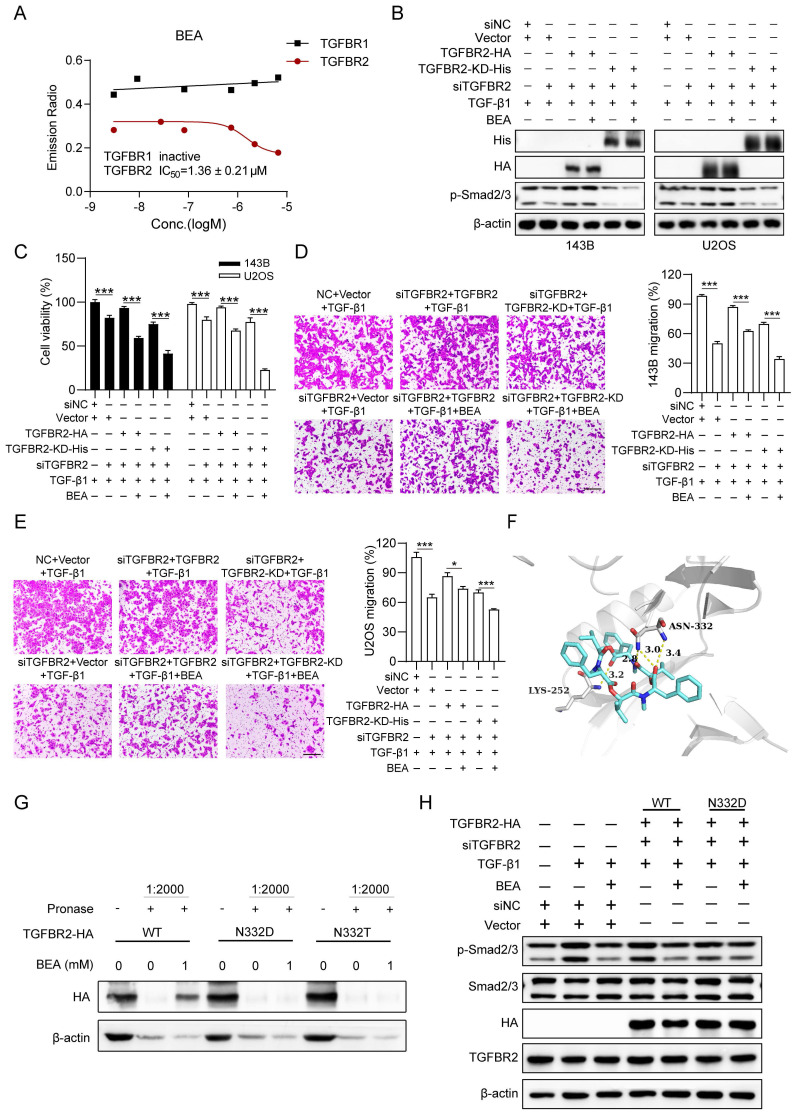
** Modes and sites of binding between BEA and TGFBR2.** (A) An *in vitro* kinase activity assay was performed to determine the effect of BEA on the activity of TGFBR2 and TGFBR1. (B-E) TGFBRII-silenced OS cells were transfected with TGFBRII-WT or TGFBR2-KD for 24 h and were then treated with TGF-β1 in the absence or presence of BEA. (B) The levels of p-Smad2/3 and Smad2/3 in OS cells was evaluated by Western blotting. (C) Cell viability in each group was determined by an MTT assay. (D-E) The migration of OS cells was assessed by a Transwell migration assay. (F) The binding modes and sites of the interaction of BEA with TGFBR2 (PDB: 5E8Y) were predicted with AutoDock Vina software. (G) The interactions of BEA with TGFBR2 mutants in OS cells were determined by DARTS approach. (H) TGFBR2-depleted OS cells were transfected with wild-type or mutant TGFBR2 for 24 h and were further treated with TGF-β1 in the absence or presence of BEA. Then, the level of p-Smad2/3 in each group was determined by Western blotting. Scale bar: 200 μm. Data are presented as mean ± SEM. *n* = 3. ^***^ *p* < 0.001.

To investigate how BEA interacts with TGFBR2 to inhibit the activation of TGF-β pathway in OS cells, the effect of BEA on the kinase activity of TGFBR2 was determined. Notably, BEA treatment potently inhibited the kinase activity of TGFBR2 with an IC_50_ value of 1.36 ± 0.21 μM, whereas BEA had a negligible suppressive effect on TGFBR1 kinase activity at concentrations of over 10 μM (**Figure 5A**). To evaluate whether the antineoplastic effect of BEA on OS cells is associated with the kinase activity of TGFBR2, OS cells with TGFBR2 knockdown were transfected with either the wild-type TGFBR2 overexpression plasmid/construct (TGFBR2) or kinase dead TGFBR2-KD plasmid/construct (**Figure S5A and S6A**). Remarkably, TGF-β1 failed to promote the phosphorylation of Smad2/3 in TGFBR2-KD-transfected OS cells compared with TGFBR2-transfected and control cells. BEA treatment reduced Smad2/3 phosphorylation in OS cells transfected with TGFBR2 or TGFBR2-KD (**Fig 5B and S6B**). Consistent with this finding, BEA treatment abrogated the TGF-β1-mediated increases in viability (**Figure 5C**) and migration (**Figure 5D-E**) of TGFBR2- and TGFBR2-KD-transfected OS cells (**Figure 5C-E**). These results suggest that BEA inhibits TGFBR2 kinase activity* in vitro* and in OS cells.

Modes and sites of the binding interaction between BEA and TGFBR2 were further predicted and analyzed by AutoDock Vina software. Our results revealed two predicted hydrogen bonds between BEA and Asn 332 (N332) and Lys 252 (K252) of TGFBR2 (**Figure 5F**). Next, OS cells were transfected with TGFBR2 overexpression plasmids containing different mutations (N332D, N332T, and K252R), and DARTS approach was employed. We found that BEA could not bind to TGFBR2^N332D^ or TGFBR2^N332T^ and failed to protect these TGFBR2 mutants (N332D and N332T) from pronase-mediated degradation in OS cells (**Fig 5G**). However, BEA suppressed the pronase-induced degradation of TGFBR2^K252R^ (**Figure S7A-B**), indicating that BEA may bind to TGFBR2 at N332 rather than K252. Furthermore, to confirm whether the binding between BEA and TGFBR2 contributes to BEA-mediated inhibition of the TGF-β pathway, TGFBR2-silenced OS cells were further transfected with wild-type TGFBR2 (TGFBR2^WT^) or the TGFBR2 N332D mutant. Our results showed that BEA treatment significantly reduced TGF-β1-induced Smad2/3 phosphorylation in TGFBR2^WT^-overexpressing OS cells, but not in TGFBR2^N332D^-transfected 143B cells (**Fig 5H and S7C**). Moreover, BEA treatment dramatically decreased the mRNA expression levels of collagen deposition-associated genes in OS cells, whereas these decreases were partially diminished by expression of the N332D mutant (**Figure S7D**). Together, our results suggest that the direct binding of BEA to Asn332 of TGFBR2 may contribute to inhibition of TGFBR2 kinase activity and Smad2/3 activation in OS cells.

### BEA suppresses the proliferation and pulmonary metastasis of OS cells *in vivo*

**Figure 6:**
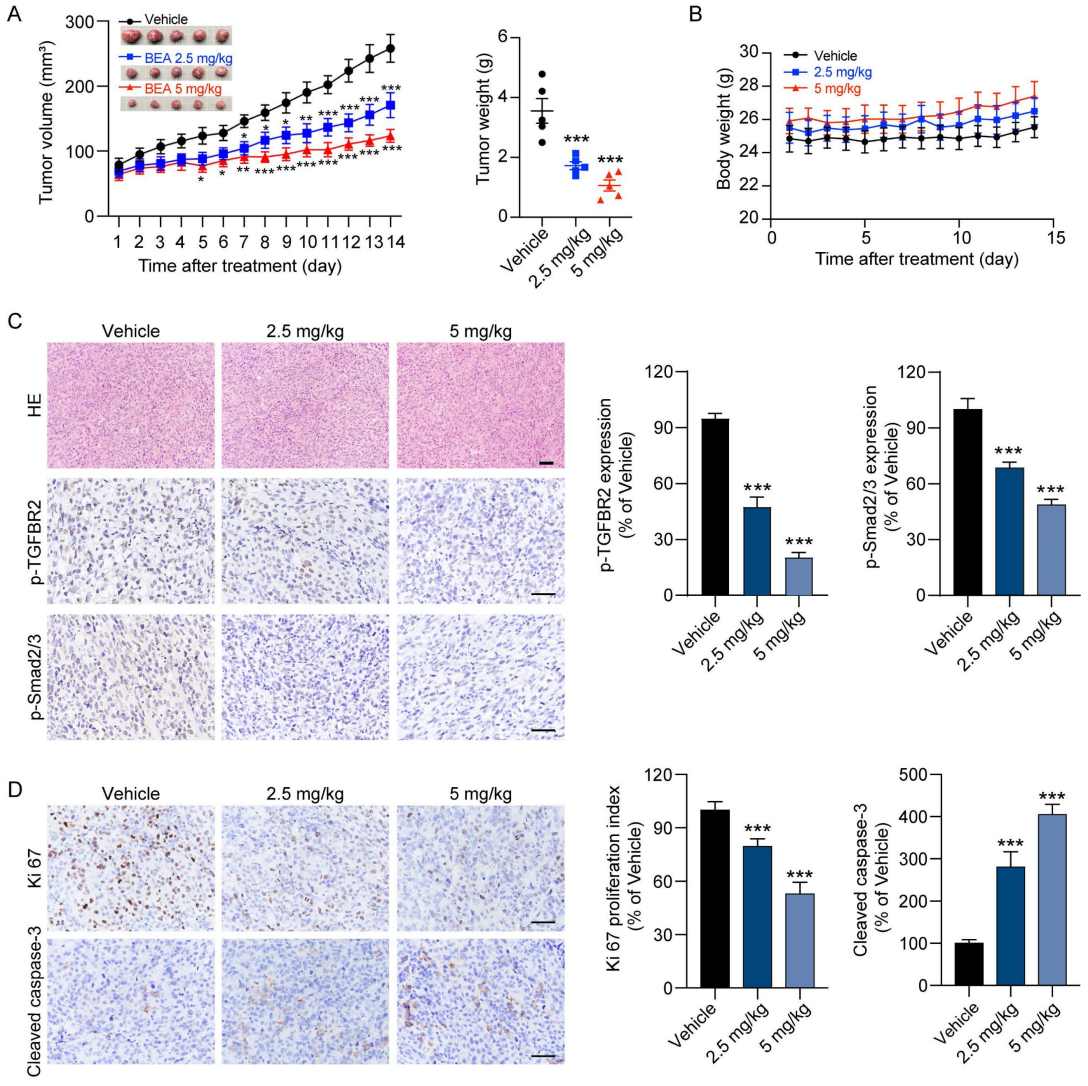
** BEA suppresses the growth of OS xenograft tumors.** (A-B) BALB/c nude mice bearing 143B xenograft tumors were treated with vehicle or BEA via intravenous injection every day for 14 days. The tumor volume and body weight of mice were evaluated every day. 143B xenograft tumors were removed and weighed. (C-D) IHC staining of (C) p-TGFBR2 and p-Smad2/3, and (D) Ki67 and Cleaved caspase-3 in 143B xenograft tumors in each group. Scale bar for H&E staining images: 100 μm. Scale bar for IHC staining images: 50 μm. Representative images and quantification of IHC staining are shown. Data are presented as mean ± SEM. *n* = 5 mice per group. ^***^ *p* < 0.001 *vs.* the vehicle group.

Finally, to investigate the effect of BEA on the proliferation and lung metastasis of OS cells* in vivo* by inhibiting the TGF-β pathway, mice bearing orthotopic 143B xenograft tumors were treated with BEA via intravenous injection. Our results showed that BEA treatment significantly suppressed the growth of 143B xenograft tumors, and that the tumor weight in the BEA-treated groups was much decreased compared with that in the vehicle group (**Figure 6A**). There was no significant loss of body weight in BEA-treated tumor-bearing mice (**Figure 6B**). H&E staining further showed that BEA treatment showed no significant toxicity in the heart, liver, spleen, lungs, and kidneys in mice (**Figure S8A**), serum concentrations of CRE and BUN in each group were not significantly changed as well (**Figure S8B**), indicating a low toxicity of BEA in tumor-bearing mice. The expression levels of p-TGFBR2 and p-Smad2/3 were significantly decreased in 143B tumors from the BEA treatment groups compared with tumors from the vehicle group (**Figure 6C**), and BEA treatment negligibly affected the level of TGF-β1 in OS xenograft tumors (**Figure S9**), which suggested that BEA treatment suppressed the TGF-β/TGFBR2/Smad2/3 pathway of OS cells *in vivo*. Additionally, the IHC staining results showed that BEA treatment significantly decreased the number of Ki67-positive proliferative cells but increased the number of cleaved caspase 3-positve apoptotic cells in OS xenograft tumors (**Figure 6D**).

**Figure 7:**
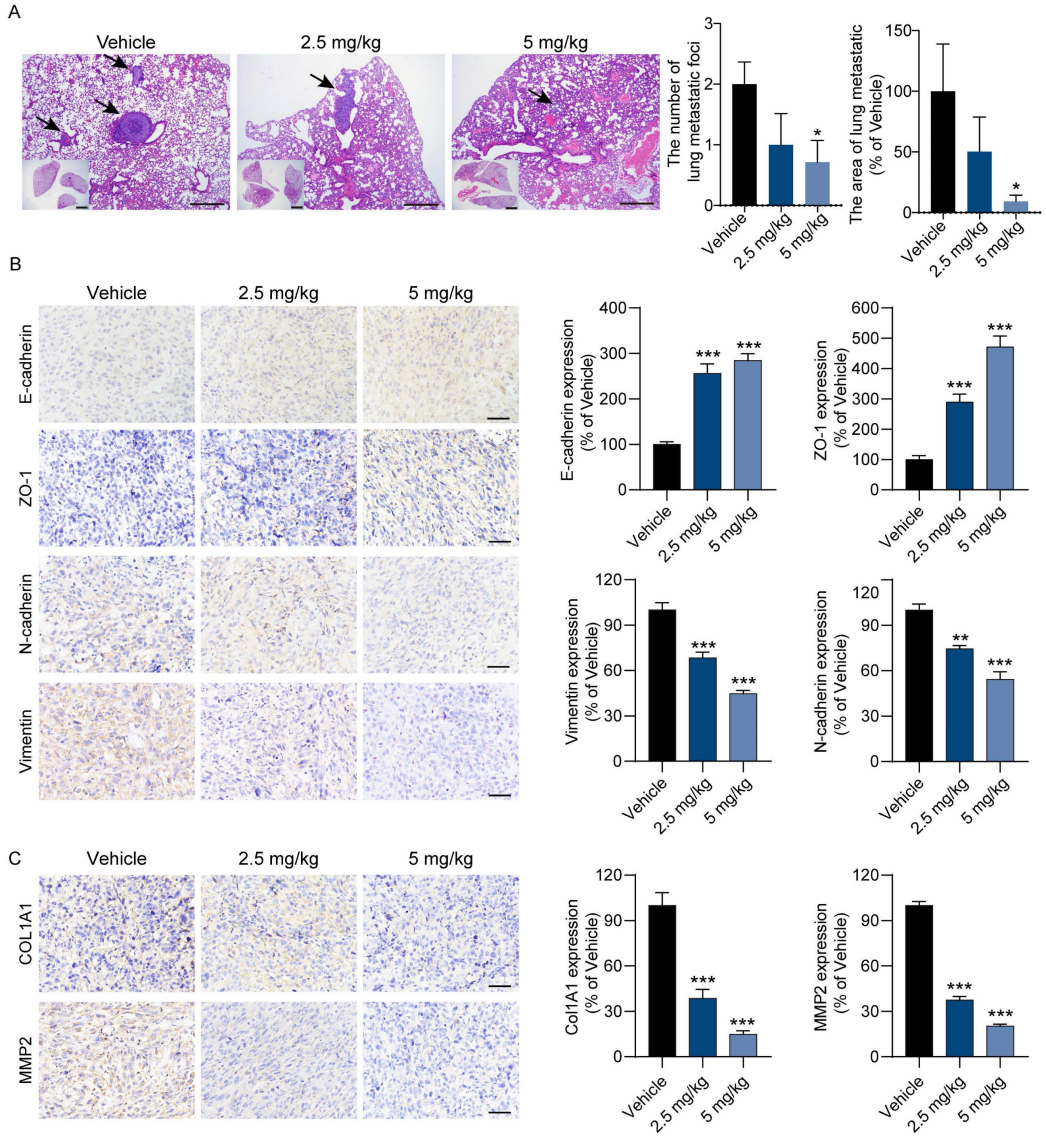
** BEA reduces the pulmonary metastasis of OS *in vivo*.** BALB/c nude mice bearing 143B xenograft tumors were treated with vehicle or BEA via intravenous injection every day for 14 days. (A) Lung tissues were harvested and subjected to H&E staining. Scale bars: 500 μm for low-magnification images and 100 μm for high-magnification images. Quantification of the area and number of lung metastatic foci in each group is shown. (B) IHC staining and expression quantification of epithelial and mesenchymal markers (E-cadherin, ZO-1, N-cadherin, and Vimentin) in 143B xenograft tumors. Scale bar: 50 μm. Quantification of IHC staining is shown. (C) IHC staining of COL1A1 and MMP2 in 143B xenograft tumors from mice treated as indicated. Scale bar: 50 μm. Quantification of IHC staining is shown. Data are presented as mean ± SEM. *n* = 5 mice per group. ^*^ p < 0.05, ^**^ p < 0.01, ^***^ p < 0.001 *vs.* the vehicle group.

Next, we also assessed the antimetastatic activity of BEA against OS cells* in vivo*. Consistent with the above findings, when compared with vehicle treatment, BEA treatment reduced the number and area of lung metastatic foci formed by 143B cells in mice (**Figure 7A**). The expression levels of mesenchymal markers and collagen deposition-associated proteins were evaluated. We found that BEA treatment markedly upregulated the levels of E-cadherin and ZO-1 but reduced the expression of N-cadherin and Vimentin in OS xenograft tumors (**Figure 7B**). The expression of COL1A1 and MMP2 was also significantly decreased in BEA-treated OS tumors (**Figure 7C**). Collectively, these results demonstrate that BEA inhibits the TGF-β pathway and suppresses the proliferation and pulmonary metastasis of OS cells *in vivo*.

## Discussion

OS is an aggressive malignancy of bone in children and adolescents, and resection in combination with adjuvant chemotherapy is still the main therapeutic approach for OS 38. Most OS patients can derive great survival benefits from chemotherapeutic agents, mainly methotrexate, doxorubicin, doxorubicin, and cyclophosphamide. However, high toxicities, severe side effects, and drug resistance readily and frequently occur during the treatment period 2, 39, 40. Concerningly, advanced, recurrent, or metastatic OS remains challenging to cure or even alleviate. These abovementioned traditional chemotherapeutic drugs are cytotoxic agents and may exert a modest effect on inhibiting the metastatic progression of OS cells triggered by various oncogenes, leading to poor prognosis. Therefore, effective therapeutic strategies for metastatic OS are urgently needed. In this study, we found that BEA, an active ingredient of traditional Chinese medicine *Bombyx batryticatus*, effectively inhibited the aggressive progression of OS cells, with low toxicity in mice bearing metastatic OS. BEA suppressed the proliferation and metastatic spread of OS cells through blockade of the TGF-β1/TGFBR2/Smad2/3 pathway. These findings demonstrated that BEA may serve as an effective and promising agent for the treatment of metastatic OS.

The TGF-β/Smad signaling pathway is frequently upregulated in multiple types of malignancies including OS and is critically associated with tumor growth, metastasis, drug resistance, and immune escape. Many natural compounds have been shown to inhibit the proliferation and metastasis of OS cells by targeting various signaling pathways 41 such as TGF-β/Smad2/3 signaling 42, 43. However, most studies have focused on whether these compounds inhibit the activation of TGF-β1-mediated Smad2/3 and enhancement of mesenchymal phenotype in OS cells and suppresses their proliferation and lung metastasis, whereas their concrete molecular targets are largely unknown. Herein, we found that BEA, a cyclic hexadepsipeptide, potently inhibited proliferation and metastasis* in vitro* and in an OS xenograft mouse model by suppressing the TGF-β1/Smad2/3 signaling, effects that may be attributed to BEA-mediated inhibition of TGFBR2 kinase activity. To block the TGF-β signaling *in vivo*, genetic ablation of *Tgfbr2* is more commonly used than genetic depletion of *Tgfbr1*, *Smad2*, and *Smad3* 21-23, indicating that TGFBR2 inhibition may have a stronger ability to suppress the TGF-β signaling. However, most TGF-β signaling inhibitors are selective TGFBR1/ALK5 inhibitors, whereas effective and selective TGFBR2 inhibitors are still scarce. In the present study, BEA inhibited the kinase activity of TGFBR2, but not TGFBR1, *in vitro*. CETSA, DARTS approach, an MST assay, and a molecular docking study further confirmed the direct binding between BEA and Asn332 of TGFBR2. Thus, our study identified an effective and selective TGFBR2 inhibitor with cyclic hexadepsipeptide structure that was distinct from that of the currently known TGF-β pathway inhibitors. Moreover, since the TGF-β pathway regulates the development and progression of various diseases, such as fibrotic diseases and immunotherapeutic resistance, we can further investigate the therapeutic efficacy of BEA against these diseases, and the findings may expand the therapeutic indications of BEA and increase the potential of BEA as a therapeutic agent.

In addition to EMT, the TGF-β/Smad pathway is also implicated in tissue fibrosis, in which ECM remodeling, which is generally characterized by increased collagen deposition, is one of the most predominant features 44. Studies have shown that upregulation of collagen components, which is mainly regulated by TGF-β1, is associated with enhanced metastasis in many types of cancer 45, 46 including OS 47. In this study, BEA treatment abrogated the TGF-β1-induced upregulation of various genes associated with collagen matrix remodeling in OS cells. BEA also inhibited the TGF-β1- and TGFBR2-mediated increases in the protein levels of COL1A1, COL3A1, MMP2, and p-FAK in OS cells and decreased the levels of COL1A1 and MMP2 in OS xenograft tumors, leading to decreased lung metastasis of OS cells. Our findings suggested that the TGF-β-induced elevated expression of collagen-related signaling molecules may be associated with increased OS metastasis, and this possibility requires further investigation. Our study also provided a perspective regarding the important role of TGF-β signaling in OS progression, which is probably mediated by promotion of mesenchymal phenotype as well as collagen deposition.

## Conclusion

In summary, this study demonstrates that BEA exerts effective antiproliferative and antimetastatic effects on OS cells *in vitro* and in mouse models. Notably, BEA directly binds to TGFBR2 and suppresses the activation of TGF-β/Smad2/3 pathway, which inhibits the proliferation, invasiveness, mesenchymal phenotype, ECM remodeling, and pulmonary metastasis of OS cells. Our findings further elucidate the implications and mechanisms of the TGF-β/TGFBR2/Smad2/3 pathway in the aggressive progression of OS and provide a rationale for evaluating BEA as a promising therapeutic agent in OS patients, especially those with pulmonary metastasis.

## Supplementary Material

Supplementary figures and tables.

## Abbreviations

OS: Osteosarcoma
BEA: Beauvericin
TGF-β: Transforming growth factor-β
EMT: Epithelial-mesenchymal transition
ECM: Extracellular matrix
FASP: Filter-aided sample preparation
siRNA: Small interfering RNA
TR-FRET: Time-resolved fluorescence resonance energy transfer
DARTS: Drug affinity responsive target stability
CETSA: Cellular thermal shift assay
MST: Microscale thermophoresis
H&E: Hematoxylin-eosin staining
IHC: Immunohistochemical staining
SEM: Standard error of mean
CRE: Creatinine
BUN: Blood urea nitrogen
