# Supplementary material for: Beauvericin suppresses the proliferation and pulmonary metastasis of osteosarcoma by selectively inhibiting TGFBR2 pathway
Source: Int J Biol Sci. 2023 Aug 21;19(14):4376–92. doi: 10.7150/ijbs.86214 (PMC10535710; doi:10.7150/ijbs.86214)
Supplement: Supplementary file 1 — Supplementary figures and tables. [file ijbsv19p4376s1.pdf]

Supplementary materials for

Beauvericin suppresses the proliferation and pulmonary metastasis of osteosarcoma by selectively inhibiting TGFBR2 pathway

Authors: Geni Ye[1,2,a], Yubo Jiao[1,2,a], Lijuan Deng[3,a], Minjing Cheng[1,2], Sheng Wang[1,2], Junqiu Zhang[1,2], Jie Ouyang[1,2], Yong Li[1], Yuxin He[2], Zhengchao Tu[2], Zhen Wang[2], Xiaojuan Song[2], Chenran Wang[1,2], Qi Qi[4], Dongmei Zhang[1,2], Lei Wang[1,2,*], Maohua Huang[1,2,*], Wencai Ye[1,2,*], Minfeng Chen [1,2,5,*].

Affiliations:

[1] State Key Laboratory of Bioactive Molecules and Druggability Assessment, Jinan University, Guangzhou, 510632, China

[2] Guangdong Province Key Laboratory of Pharmacodynamic Constituents of Traditional Chinese Medicine and New Drugs Research, College of Pharmacy, Jinan University, Guangzhou, 510632, China

[3] Guangzhou Key Laboratory of Formula-Pattern of Traditional Chinese Medicine, Jinan University, Guangzhou, 510632, China

[4] MOE Key Laboratory of Tumor Molecular Biology, Clinical Translational Center for Targeted Drug, Department of Pharmacology, School of Medicine, Jinan University, Guangzhou, 510632, China.

[5] State Key Laboratory of Chemical Oncogenomics, Guangdong Provincial Key Laboratory of Chemical Biology, Tsinghua Shenzhen International Graduate School, Shenzhen, 518055, China

[a] These authors contributed equally to this study.

* Corresponding authors: Minfeng Chen, E-mail: minfengchen@jnu.edu.cn. Wencai Ye, E-mail: chywc@aliyun.com. Maohua Huang, E-mail: mhhuang@jnu.edu.cn. Lei Wang, E-mail: cpuwanglei@126.com.

Supplementary figures and figure legends

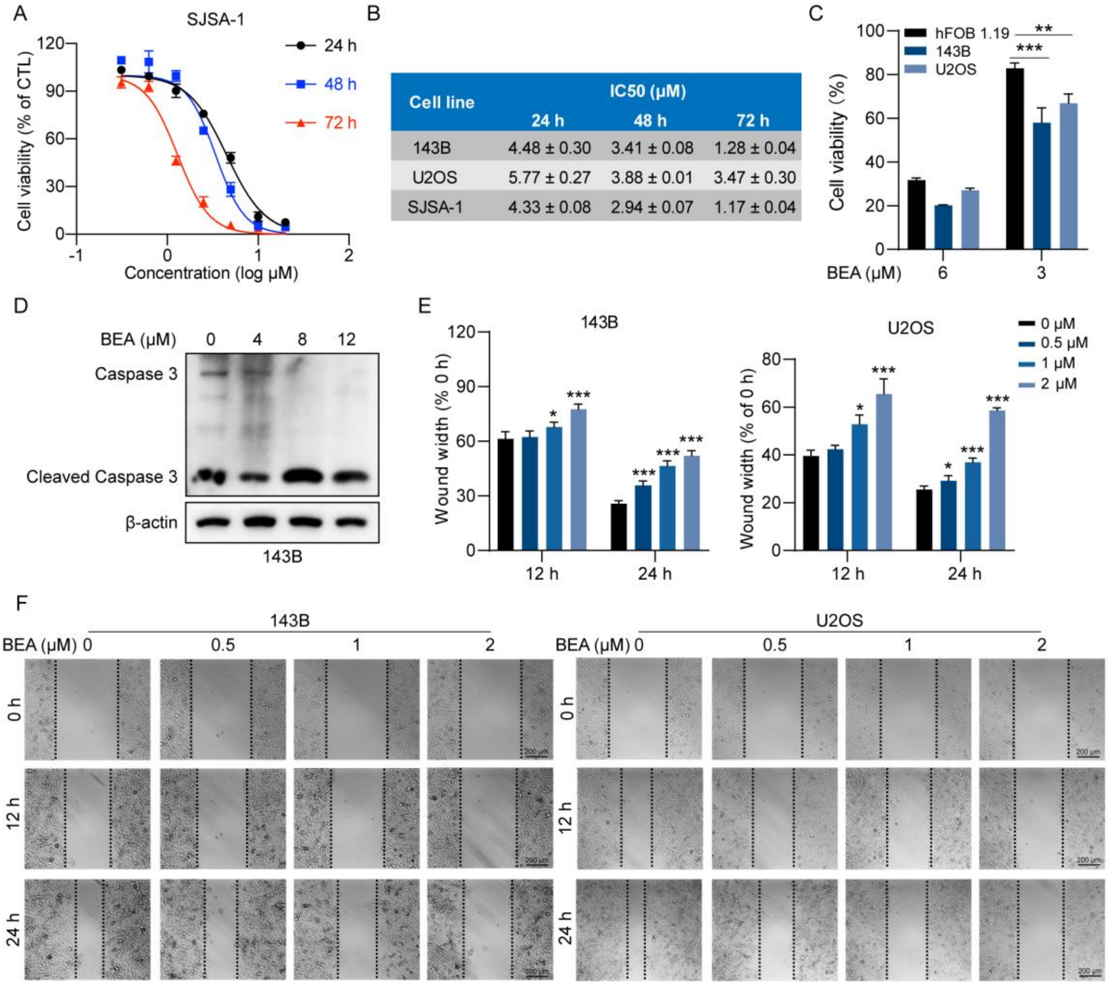

Figure S1 BEA inhibits the viability and migration of OS cells. (A) The effect of BEA on the viability of SJSA-1 cells was evaluated by an MTT assay. (B) A table showing the IC_{50} values of BEA in 143B, U2OS, and SJSA-1 cells. (C) An MTT assay was performed to evaluate the viability of hFOB 1.19 osteoblasts and 143B and U2OS cells treated with BEA (3 and 6 μM). Data are presented as mean ± SEM. $n = 3$. ** $p < 0.01$ and *** $p < 0.001$. (D) 143B cells were treated with BEA (4, 8, and 12 μM) for 24 h, and the expression of Caspase-3 and cleaved Caspase-3 was evaluated by Western blotting. (E and F) Representative images and quantification of wound healing assay. Scale bar: 200 μm. Data are presented as mean ± SEM.

$n = 3$. $^{*}p < 0.05$ and $^{***}p < 0.001$ *vs.* the CTL (control) group.

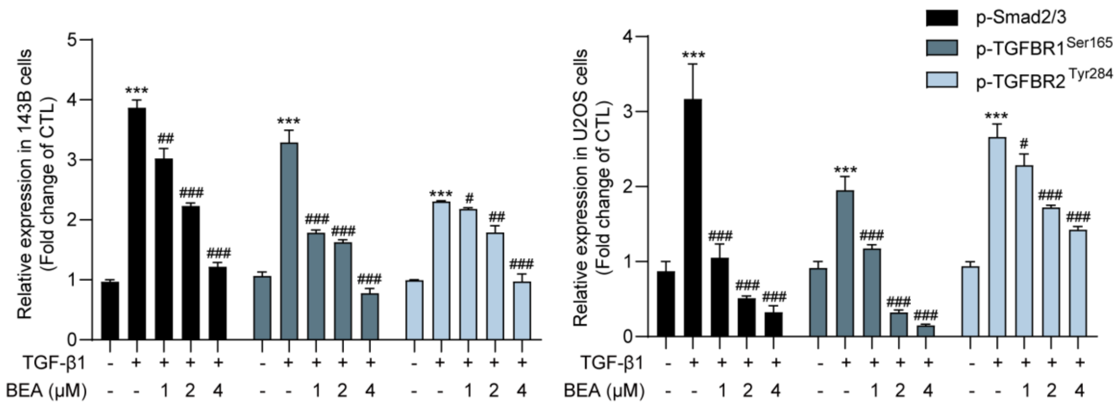

Figure S2 BEA inhibits the TGF-β/Smad2/3 signaling pathway in OS cells. 143B and U2OS cells treated with BEA for 24 h, and the expression of p-Smad2/3, p-TGFBR1 (Ser165), and p-TGFBR2 (Tyr284) in OS cells were determined by Western blotting. Quantification of the blots in Figure 2B is shown. Data are presented as mean ± SEM. $n = 3$. $^{***}p < 0.001$ *vs.* the CTL (control) group. $^{\#}p < 0.05$, $^{\#\#}p < 0.01$, and $^{\#\#\#}p < 0.001$ *vs.* the TGF-β1-treated group.

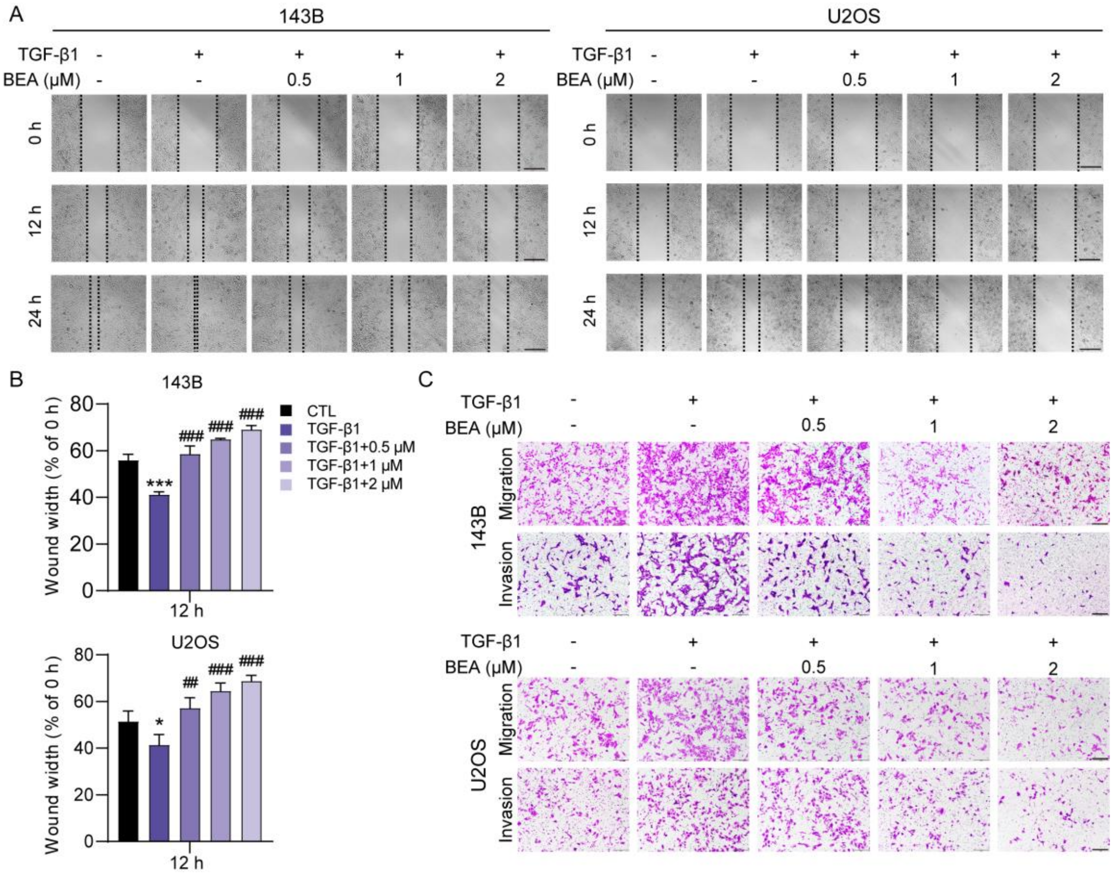

Figure S3 BEA suppresses TGF-β1-induced migration and invasion in OS cells. (A and B) The effect of BEA on the migration of OS cells was detected by wound healing assay. Representative images and quantification of wound width at 12 h are shown. Scale bar: 200 μm. (C) Transwell migration and invasion assays were performed to evaluate the effect of BEA on the TGF-β1-induced migration and invasion of 143B and U2OS cells. Representative images are shown. Scale bar: 200 μm. Data are presented as mean ± SEM. n = 3. * $p < 0.05$ and *** $p < 0.001$ *vs.* the CTL (control) group. $^{\#\#}$ $p < 0.01$ and $^{\#\#\#}$ $p < 0.001$ *vs.* the TGF-β1-treated group.

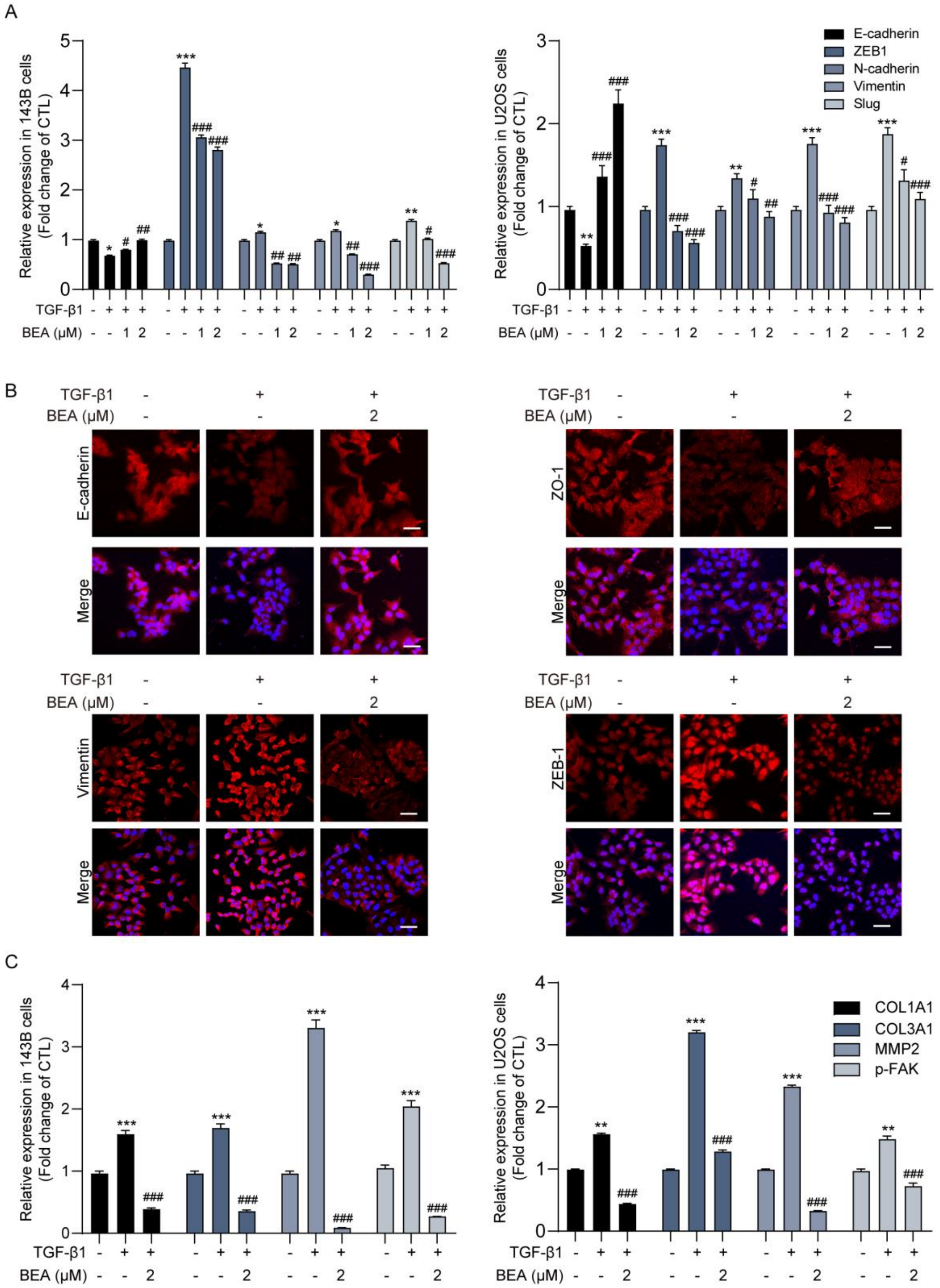

Figure S4 BEA inhibits TGF-β1-induced mesenchymal phenotype in OS cells. 143B and U2OS cells were treated with BEA for 48 h and the expression of E-cadherin, ZEB1, N-cadherin, Vimentin, Slug, COL1A1, COL3A1, MMP2 and p-FAK(Tyr397) in OS cells were determined by Western blotting. (A) Quantification of the blots in Figure 3H is shown. (B)

143B cells were treated with BEA for 48 h. Representative images of immunofluorescence staining of E-cadherin, ZO-1, Vimentin, and ZEB1 in OS cells treated with BEA and TGF-β1. Scale bar: 50 μm. (C) Quantification of the blots in Figure 3I is shown. Data are presented as mean ± SEM. $n = 3$. * $p < 0.05$, ** $p < 0.01$, and *** $p < 0.001$ *vs.* the CTL (control) group. $^{\#}$ $p < 0.05$, $^{\#\#}$ $p < 0.01$, and $^{\#\#\#}$ $p < 0.001$ *vs.* the TGF-β1-treated group.

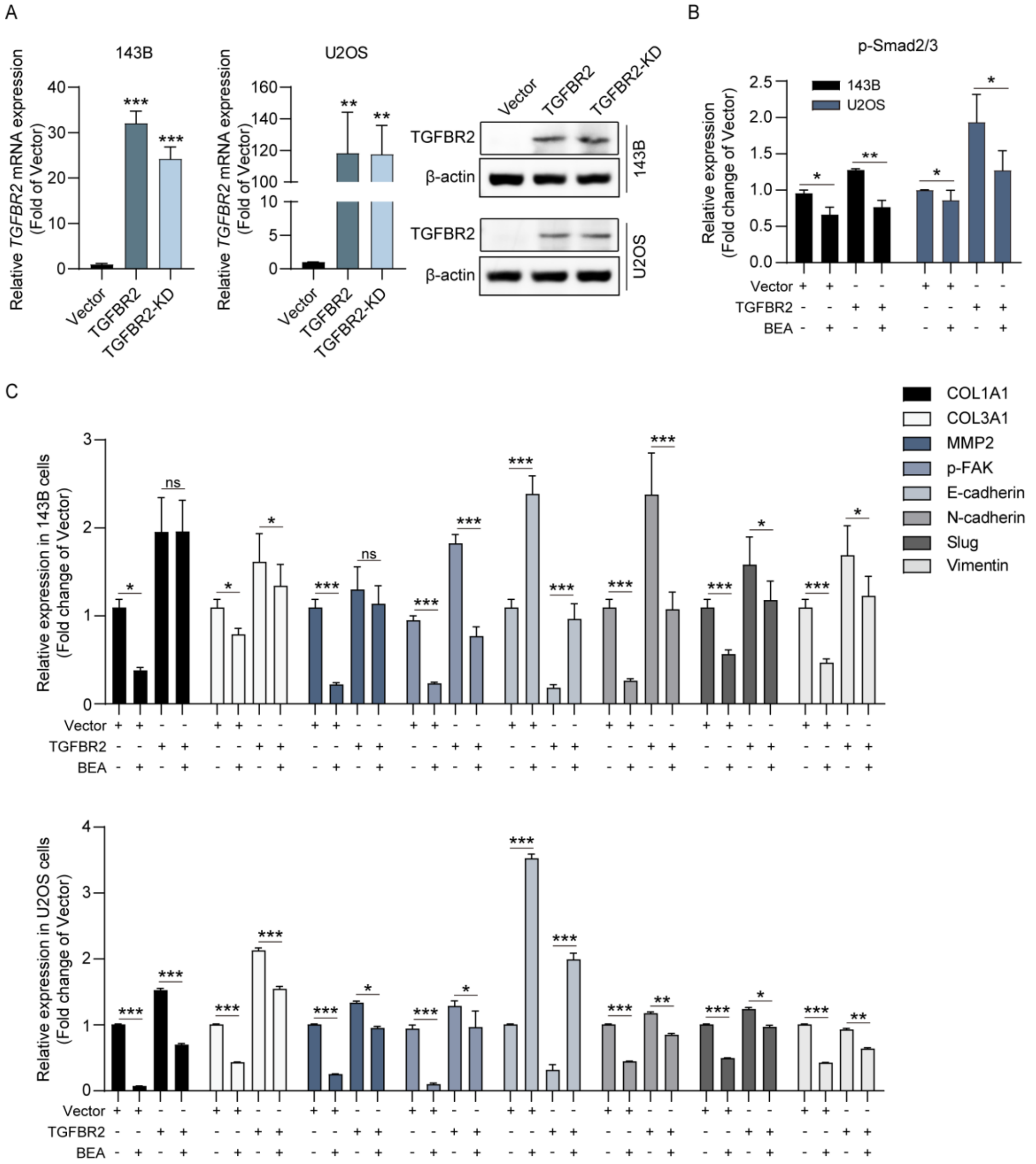

Figure S5 BEA inhibits Smad2/3 phosphorylation and suppresses mesenchymal

phenotype in TGFBR2-overexpressing OS cells. (A) OS cells were transfected with TGFBR2 and TGFBR2-KD and their corresponding vectors. qPCR and Western blotting were conducted to evaluate the mRNA and protein levels of TGFBR2 in OS cells. Data are presented as mean ± SEM. $n = 3$. ** $p < 0.01$ and *** $p < 0.001$ *vs*. the vector-transfected OS cells. (B) Quantification of the blots in Figure 4E is shown. (C) Quantification of the blots in Figure 4H is shown. Data are presented as mean ± SEM. $n = 3$. * $p < 0.05$, ** $p < 0.01$, and *** $p < 0.001$.

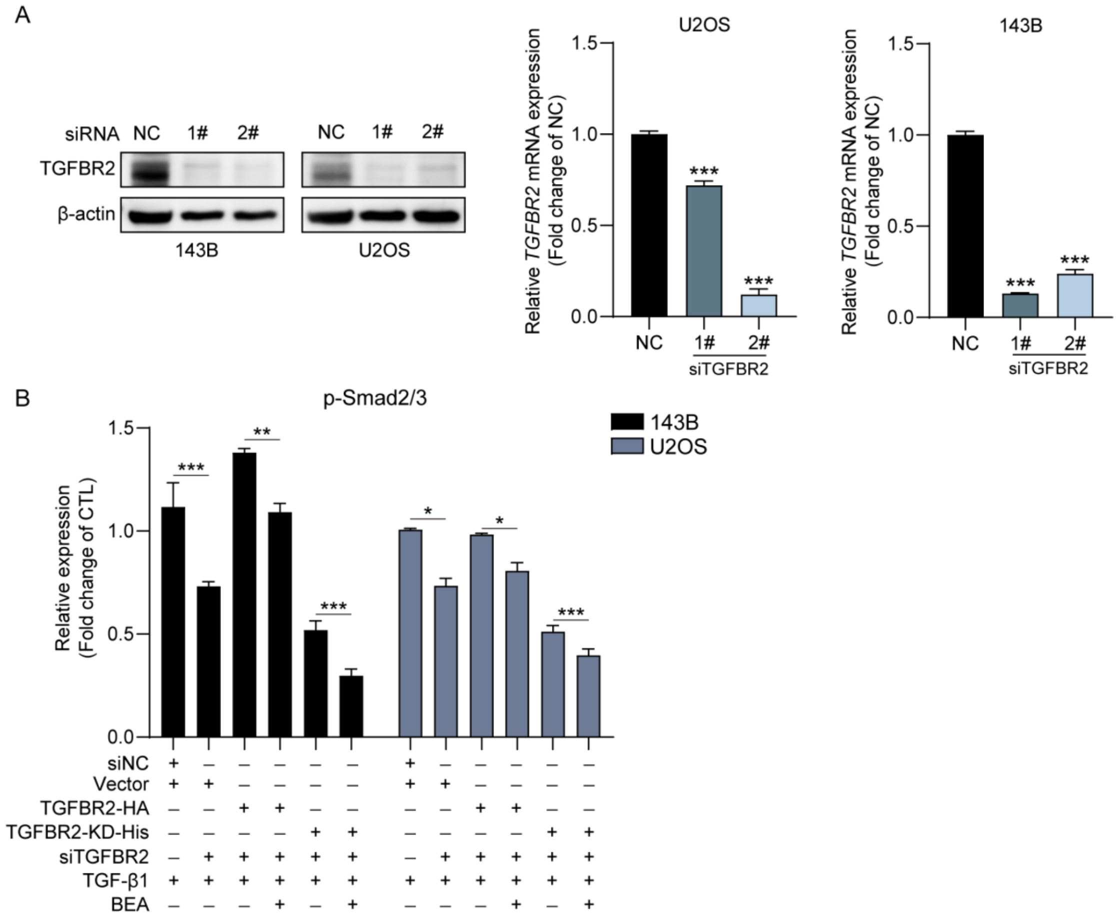

Figure S6 BEA inhibits Smad2/3 phosphorylation in OS cells. (A) OS cells (143B and U2OS) were transfected with NC or TGFBR2 siRNA. The mRNA and protein levels of TGFBR2 in OS cells were determined by qPCR and Western blotting. NC, negative control.

Data are presented as mean ± SEM. $n = 3$. $^{***}p < 0.001$ *vs.* the OS cells transfected with NC siRNA. (B) Quantification of the blots in Figure 5B is shown. Data are presented as mean ± SEM. $n = 3$. $^{**}p < 0.01$ and $^{***}p < 0.001$. ns, no significance.

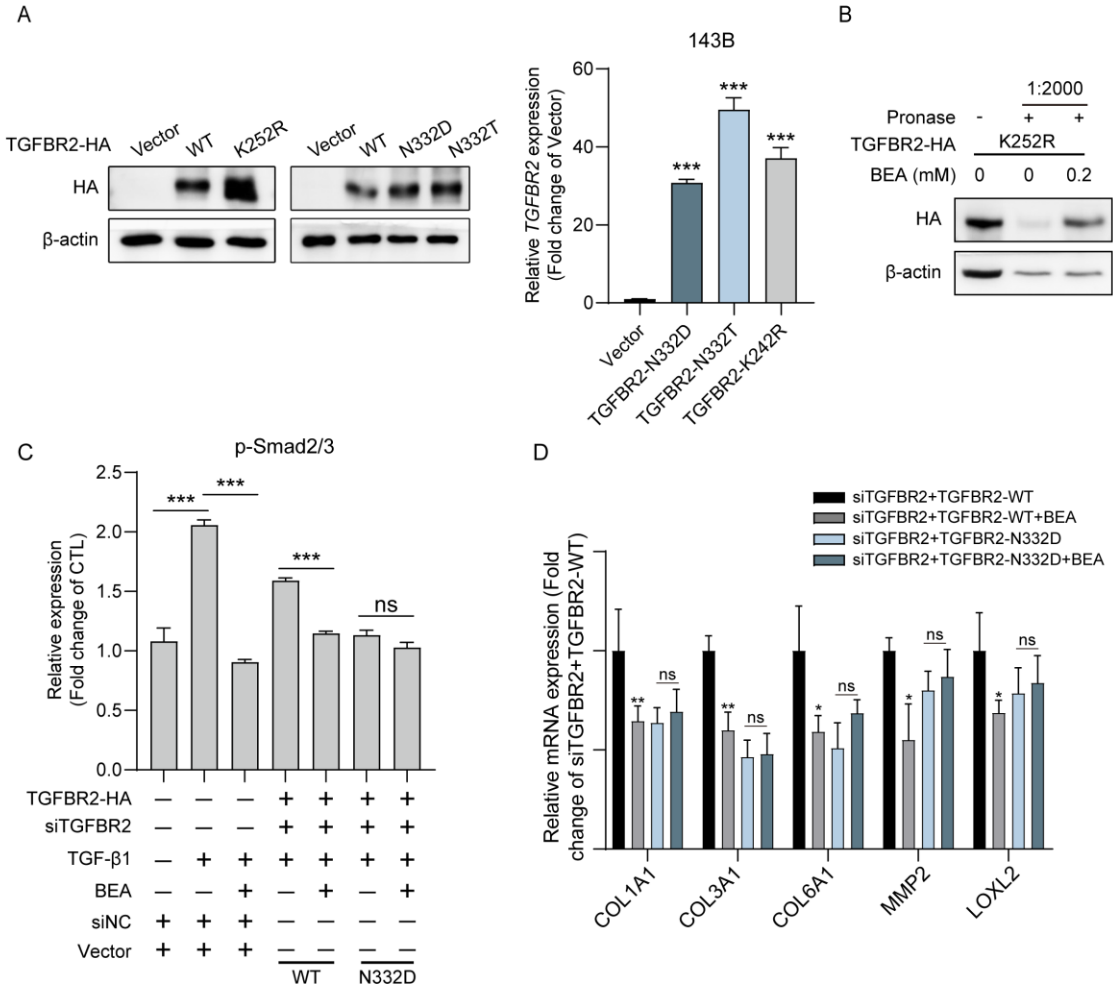

Figure S7. BEA negligibly affects Smad2/3 phosphorylation and the expression of collagen deposition-associated genes in OS cells transfected with mutant TGFBR2. (A) 143B cells were transfected with wild-type or mutant TGFBR2 (K252R, N332D, and N332T) and their corresponding vectors. qPCR and Western blotting were conducted to evaluate the mRNA and protein levels of TGFBR2 in OS cells. Data are presented as mean ± SEM. $n = 3$. $^{***}p < 0.001$ *vs.* the Vector group. (B) The effect of BEA on the stabilization of TGFBR2

(K252R) in 143B cells was determined by DARTS approach. (C) Quantification of the blots in Figure 5H is shown. Data are presented as mean ± SEM. n = 3. $^{***}P < 0.001$. ns, no significance. (D) TGFBR2-depleted OS cells were co-transfected with wild-type or mutant TGFBR2 for 24 h, and collagen deposition-associated proteins in OS cells after treatment with BEA were determined by qPCR. Data are presented as mean ± SEM. n = 3. $^{*}p < 0.05$ and $^{**}p < 0.01$ *vs.* the siTGFRB2+TGFBR2-WT group. ns, no significance.

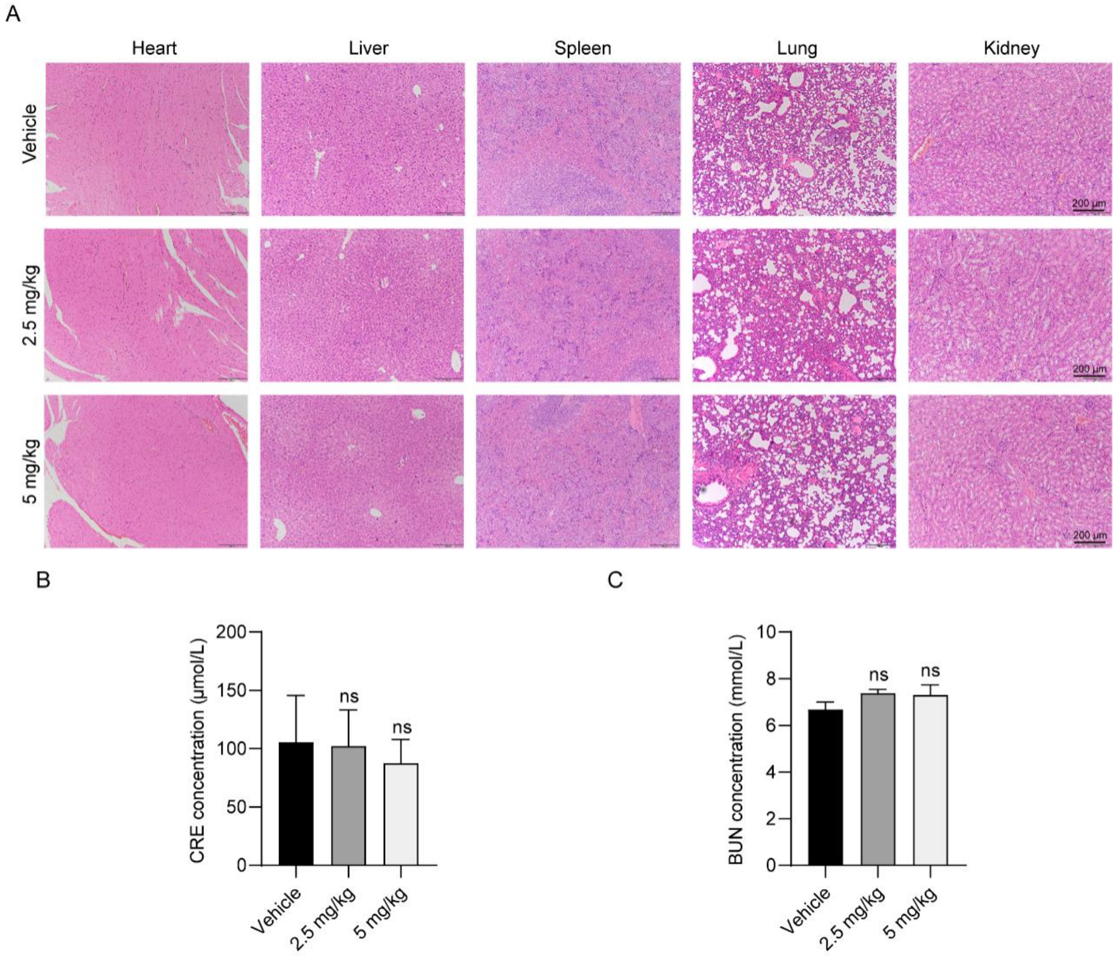

Figure S8 BEA shows low toxicity in 143B tumor-bearing mice. (A) H&E staining of the heart, liver, spleen, lung, and kidney of tumor-bearing mice treated with vehicle or BEA. Scale bar: 200 μm. (B) ELISA analysis of the serum concentrations of CRE and BUN in tumor-bearing mice from each group. Data are presented as mean ± SEM. n = 5 mice per

group. ns, no significance *vs.* the vehicle group.

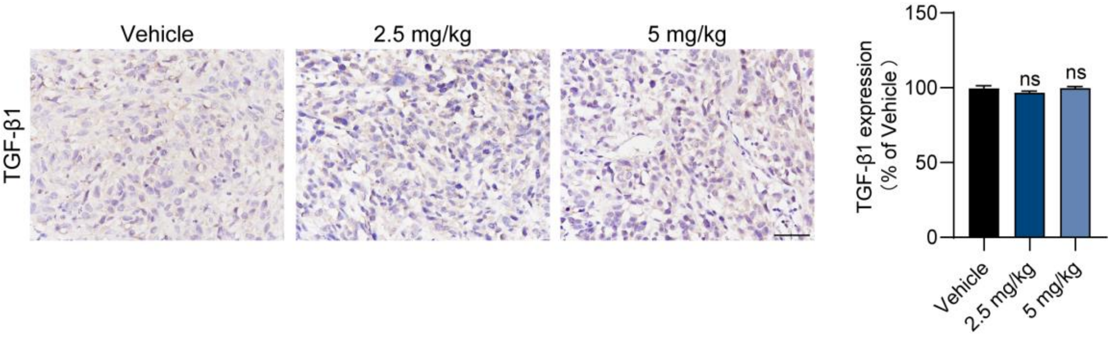

Figure S9 BEA negligibly affects the level of TGF-β1 in OS xenograft tumors. BALB/c nude mice bearing 143B xenograft tumors were treated with vehicle or BEA via intravenous injection every day for 14 days. IHC staining of TGF-β1 in each group. Scale bar: 50 μm. Representative images and quantification of IHC staining are shown. Data are presented as mean ± SEM. *n* = 5 mice per group. ns, no significance.

Supplementary Tables

Supplementary Table 1. Primer sequences used for qPCR.

Gene name	Forward/Reverse	Sequence 5’ to 3’
CDH1	Forward	TCTCTCACGCTGTGTCATCC
(E-cadherin)	Reverse	CACTGGATTTGTGGTGACGA
Vim	Forward	GTCCGTGTCCTCGTCCTCCTAC
(Vimentin)	Reverse	AGTTGGCGAAGCGGTCATTCAG
CDH2	Forward	AGGCGTCTGTAGAGGCTTCTGG
(N-cadherin)	Reverse	TCTGCTGACTCCTTCACTGACTCC
SNAI2	Forward	CCTGGTCAAGAAGCATTTCAACGC
(Slug)	Reverse	GGAGGAGGTGTCAGATGGAGGAG
TWIST1 (Twist)	Forward	GCCGGAGACCTAGATGTCATT
	Reverse	CCCACGCCCTGTTTCTTTGA
ZEB1	Forward	CCCATTACAGGCAACCAGTTCTCC

	Reverse	GAAGTTGGCTAGGCTGCTCAAGAC
TJP1	Forward	GGCGGATGGTGCTACAAGTGATG
(ZO-1)	Reverse	AGGCTCAGAGGACCGTGTAATGG
COL1A1	Forward	TGCTGGAAACCCTGGTGCTG
	Reverse	GTTCACCTCGAGCTCCTCGC
COL3A1	Forward	TGTACCAGCCAGACCAGGAAGAC
	Reverse	TGTACCAGCCAGACCAGGAAGAC
COL6A1	Forward	CAGCTGGGCCTGCAGGATAC
	Reverse	TCTTGGGCCAGCCTCTCCAT
COL10A1	Forward	CAGCTGGGCCTGCAGGATAC
	Reverse	TCTTGGGCCAGCCTCTCCAT
MMP2	Forward	CGACCACAGCCAACTACGATGATG
	Reverse	GTGCCAAGGTCAATGTCAGGAGAG
LOX	Forward	TGGCTGAAGGCCACAAAGCA
	Reverse	TGTGCAGCCTGAGGCATACG
LOXL2	Forward	CAAGCACTGGACGGCCAAGA
	Reverse	CCAGTAGCGCTGCTTCCTCC
TGFBR1	Forward	GTTCGTGGTTCCGTGAGGCA
	Reverse	AAGATGGGCAAGACCGCTCG
TGFBR2	Forward	CACGCCAAGGGCAACCTACA
	Reverse	GATGGGCATCTTGGGCCTCC
ACTB	Forward	TCTTCCAGCCTTCCTTCCTG
	Reverse	CCTGCTTGCTGATCCACATC

Supplementary Table 2. siRNA sequences used for cell transfection.

Gene name	Sense/Antisense	Sequence 5’ to 3’
TGFBR2-homo-578 (1#)	Sense	AAGGACAUCUUCUCAGACAUC
	Antisense	GAUGUCUGAGAAGAUGUCCUU
TGFBR2-homo-729 (2#)	Sense	GCUCUGAUGAGUGCAAUGA
	Antisense	UCAUUGCACUCAUCAGAGC
